# Most Useful Acupuncture Points (MUAPs) Teaching Cluster: A Structured Narrative Review of Mechanistic Evidence for Biomedical Acupuncture Training

**DOI:** 10.7759/cureus.106511

**Published:** 2026-04-06

**Authors:** Yiangos Karavis, Miltiades Karavis

**Affiliations:** 1 Anesthesia and Critical Care, Aretaieion Hospital, Athens, GRC; 2 Rehabilitation Medicine, AcuScience, Athens, GRC

**Keywords:** acupuncture therapy, autonomic nervous system modulation, evidence-based acupoints, most useful acupuncture points, vagal pathways

## Abstract

Background: Acupuncture is increasingly evaluated within biomedical frameworks, yet its integration into modern practice remains limited by unclear mechanisms, heterogeneous training standards, and the lack of transparent point-selection systems. Traditional meridian models align poorly with contemporary neurophysiology, contributing to inconsistent protocols and barriers to rigorous research. This review examines the Most Useful Acupuncture Points (MUAPs) as a candidate, mechanism-driven acupoint cluster developed to address functional dysregulation and autonomic imbalance while providing a practical theoretical teaching framework. A structured narrative evidence synthesis was conducted using PubMed, Google Scholar, and backward citation tracking. Studies were included if they examined at least one prespecified candidate MUAP (LI4, PC6, ST36, SP6, LR3, GV20, GV26, GB34, TW5, CV6, or auricular vagus zone) and reported measurable physiological outcomes related to autonomic modulation, inflammatory signaling, neuroimmune pathways, brain-gut reflexes, limbic or cortical activity, or HRV (heart rate variability) parameters. A total of 71 studies met the inclusion criteria and underwent standardized mechanistic extraction. Across animal and human models, MUAPs demonstrated convergent effects across multiple physiological domains. ST36 and PC6 were repeatedly associated with vagal pathways, increased high-frequency HRV, and suppressed sympathetic activity. ST36, SP6, LI4, and GV20 reduced pro-inflammatory cytokines and modulated NF-κB and NLRP3 signaling. LI4, LR3, ST36, and GV20 influenced limbic-paralimbic and cortical networks. Multiple points improved visceral motility, analgesia, neuroprotection, and cognitive outcomes, with recurrent mechanistic support across studies. MUAPs represent a candidate, mechanistically grounded acupoint cluster with effects on autonomic, neuroimmune, and neurophysiological systems. This framework may support future educational and clinical protocol development, but requires prospective validation.

## Introduction and background

Acupuncture has evolved from a tradition-based medical system grounded in meridian theory into a globally practiced therapeutic modality increasingly examined through biomedical frameworks. Despite growing clinical adoption and scientific interest, its integration into modern healthcare remains limited by persistent challenges, including incomplete mechanistic explanations, heterogeneous educational standards, and the absence of reproducible, physiology-driven systems for acupoint selection [1-3]. Traditional acupuncture theory relies on cosmological constructs such as qi, meridians, and zang-fu relationships, which lack direct correspondence with contemporary understandings of neurophysiology, immunology, and systems biology, contributing to ongoing skepticism and variability in clinical application [3,4].

Acupuncture education traditionally requires trainees to learn hundreds of acupoints with limited guidance on prioritization or mechanistic relevance. This approach imposes substantial cognitive load, particularly for beginners, and offers little support for early clinical competency or evidence-based decision-making [5,6]. The lack of standardized, mechanism-driven acupoint clusters also undermines research reproducibility, contributing to heterogeneity in clinical trials and limiting the development of consensus-based protocols compatible with modern reporting standards such as CONSORT and STRICTA [7,8]. As a result, both education and research in acupuncture remain fragmented, despite increasing interest from biomedical disciplines.

In parallel, a substantial body of experimental and clinical research has begun to elucidate the biological mechanisms underlying acupuncture. Mechanistic studies demonstrate that stimulation of specific acupoints can modulate autonomic nervous system activity, alter heart rate variability (HRV), engage vagal and brainstem circuits, suppress inflammatory signaling, and influence limbic and cortical networks involved in affective regulation, pain processing, and homeostasis [9-11]. Preclinical models have shown that stimulation at points such as ST36 and PC6 activates vagal-adrenal and cholinergic anti-inflammatory pathways, providing biological explanations for their broad clinical effects across gastrointestinal, inflammatory, and stress-related conditions [12,13]. Auricular vagus nerve stimulation similarly activates central autonomic nuclei, producing neuromodulatory effects analogous to invasive vagal nerve stimulation through noninvasive means [14,15].

Importantly, the acupoints most frequently cited in classical medical texts are often the same points that recur across modern mechanistic, clinical, and network-analytic studies, suggesting that their historical prominence reflects reproducible physiological effects rather than tradition alone [16-18]. However, this convergence has not been systematically synthesized into a unified, evidence-based teaching or clinical framework.

In this manuscript, we use the term Most Useful Acupuncture Points (MUAPs) to refer to a prespecified candidate cluster of acupoints selected for targeted mechanistic evaluation. Here, “most useful” should not be interpreted as an objectively proven ranking across all acupuncture points, but as a working conceptual framework based on recurrent appearance in biomedical, mechanistic, clinical-pattern, and network-analytic literature. The aim of this review is therefore not to identify, de novo, the universally most important acupoints in acupuncture, but to synthesize the mechanistic and experimental evidence relevant to this candidate cluster, clarify the rationale for its inclusion, and evaluate its potential relevance as a theoretical framework for biomedical acupuncture education and future protocol testing.

## Review

Methods

Review Design and Scope

This manuscript was developed as a structured narrative review of mechanistic evidence. The objective was not to perform a formal systematic review of clinical effectiveness for a single disease entity, but to synthesize convergent physiological and mechanistic findings across diverse experimental and clinical study designs relevant to a prespecified candidate MUAP cluster. Because of heterogeneity across populations, model systems, stimulation modalities, comparators, and outcome measures, the review prioritized mechanistic convergence and conceptual coherence over quantitative effect-size pooling.

Literature Search Strategy

A targeted literature search was conducted in PubMed and Google Scholar, supplemented by backward citation tracking of relevant reviews and highly cited mechanistic studies. PubMed was selected for biomedical indexing and structured retrieval of peer-reviewed mechanistic and clinical literature, while Google Scholar was used to broaden citation retrieval and identify additional relevant studies not always captured through indexed database searching alone. The search was performed on November 20, 2025.

Searches used predefined combinations of acupoint, intervention, and mechanistic outcome terms. The exact executed search strategies were as follows: PubMed: ("Acupuncture Points"[Mesh] OR "Acupuncture"[Mesh] OR acupuncture OR needling)AND(ST36 OR LI4 OR LR3 OR SP6 OR GB34 OR PC6 OR TW5 OR CV6 OR GV20) AND(neuromodulation OR "vagus nerve" OR inflammation OR cytokines OR HRV OR autonomic). Google Scholar search included broad keyword combinations such as “acupuncture major points de qi”; “high centrality acupoints neuromodulation”; “ST36 inflammation vagus”; “LI4 autonomic regulation”; “SP6 HRV acupuncture”; “GV20 cortical modulation”, and “auricular vagus concha acupuncture”.

No initial date restriction was applied because the purpose was to capture both foundational and contemporary biomedical literature relevant to the candidate MUAP cluster. However, priority in interpretation was given to studies with objectively measurable physiological endpoints and clearer mechanistic relevance.

Generation of the Candidate MUAP Framework

The candidate MUAP cluster was prespecified before full-text synthesis. The initial list was derived from convergent signals across prior clinical-pattern literature, mechanistic studies, and network or data-mining studies reporting recurring centrality or repeated use of specific points in biomedical acupuncture contexts. This candidate framework was intended as a focused starting set for targeted mechanistic review rather than a definitive or exhaustive ranking of all acupuncture points. Because the search then evaluated literature involving these prespecified points, the framework should be interpreted as hypothesis-generating and potentially vulnerable to circularity and confirmation bias.

Study Selection and Eligibility Criteria

Studies were eligible if they (1) examined at least one prespecified candidate MUAP or auricular vagus site; (2) reported objectively measurable biomedical outcomes related to autonomic modulation, HRV, inflammatory or neuroimmune signaling, neuroendocrine activity, brain-gut regulation, or central nervous system function; (3) employed manual acupuncture, electroacupuncture, transcutaneous electrical stimulation, auricular stimulation, or related biologically measurable stimulation modalities; and (4) provided sufficient methodological detail to permit mechanistic interpretation.

Studies were excluded if they (1) relied exclusively on traditional meridian-based interpretation without measurable physiological endpoints; (2) focused on acupoints outside the candidate MUAP framework without directly informing the mechanistic interpretation of that framework; (3) lacked objectively measurable physiological outcomes; or (4) provided insufficient methodological detail. Both human and animal studies were eligible because the review question was mechanistic rather than disease-specific or purely clinical.

Titles, abstracts, and full texts were screened according to the above criteria. Initial screening was conducted by YK. Where eligibility was uncertain, decisions were resolved by a consensus process with YK and MK. Because the review used a targeted candidate-point approach rather than an exhaustive de novo search across all acupuncture points, the study-selection process is inherently more vulnerable to confirmation bias than a formal systematic review and should be interpreted accordingly.

Data Extraction and Synthesis

For each included study, data were extracted using a standardized framework designed to capture mechanistic relevance rather than clinical efficacy alone. Extracted variables included publication details, study design, model type (human or animal), stimulation modality, acupoints evaluated, comparator conditions, and primary physiological outcomes. Particular emphasis was placed on mechanisms relevant to MUAPs, including autonomic balance, HRV indices, vagal activation, inflammatory cytokine profiles, neuroimmune signaling pathways, cortical and limbic activity, and brainstem circuit engagement.

Evidence synthesis followed an iterative thematic approach. Findings were grouped first by candidate acupoint and then by a broader mechanistic domain, including autonomic regulation, inflammatory and neuroimmune modulation, central nervous system engagement, gut-brain interaction, pain mechanisms, and organ-level functional effects. Because the included studies were highly heterogeneous in model type, intervention modality, comparator choice, and endpoint definition, formal meta-analysis and subgroup analysis were not considered appropriate. The synthesis, therefore, emphasizes thematic mechanistic convergence rather than quantitative confirmation.

Identification of MUAPs

The current review does not establish a de novo ranking of acupuncture points across the full literature. Instead, it evaluates whether a prespecified candidate MUAP cluster shows recurrent mechanistic support across multiple biomedical domains. Candidate points were considered conceptually supported when they appeared repeatedly across independent studies and were associated with convergent findings in autonomic, HRV, inflammatory, neuroimmune, or cortical-limbic domains. Network-analytic and data-mining studies were used to contextualize, but not definitively validate, the candidate framework. Accordingly, MUAPs should be interpreted as a candidate mechanistic cluster rather than as an objectively validated hierarchy of point usefulness.

Results

Overview of Included Evidence

A total of 73 studies met the eligibility criteria and were included in the final narrative synthesis. The evidence base comprised mechanistic animal studies, controlled human physiological studies, randomized trials, and systematic or network-based analyses. Although the included literature was heterogeneous in species, stimulation modalities, comparator conditions, and modeled disorders, several candidate points, particularly ST36, PC6, LI4, LR3, SP6, GV20, and GV26, recurred across autonomic, inflammatory, neuroimmune, and central nervous system domains. These findings are presented as evidence of mechanistic convergence within a prespecified candidate framework, not as proof that these are the universally most useful points across all acupuncture. Detailed study characteristics, including model type, stimulation modality, acupoints evaluated, mechanistic domains, and primary physiological outcomes, are provided in Supplemental Material 1.

A high-level synthesis of the mechanistic domains activated by MUAPs-related stimulation, organized by autonomic, inflammatory, neuroimmune, and central nervous system pathways, is summarized in Table 1.

**Table 1:** MUAPs Mechanistic Domains and Representative Evidence MUAPs: Most Useful Acupuncture Points; CNS: central nervous system; DMV: dorsal motor vagal neurons; NTS: nucleus tractus solitarius

Mechanistic Domain	Physiological Pathways	Representative MUAPs Acupoints	Key Evidence (Representative Studies)	Overall Direction of Effect
Autonomic Nervous System (ANS)	↑ vagal efferent activity; ↓ sympathetic tone; normalization of LF/HF ratio; activation of vagovagal reflex; modulation of NTS - DMV circuits	ST36, PC6, LI4	Zhang et al. [19] (ST36): ↑ HF vagal tone, ↓ LF/HF during cold stress; Liu et al. [20] (ST36): ↑ vagal activity in rectal distension (RD) model; Lu et al. [21] (PC6): ↑ parasympathetic discharge	Strong parasympathetic activation; improved autonomic balance
Cholinergic Anti-Inflammatory Pathway (CAP)	α7nAChR activation; inhibition of cytokine release; vagal–splenic modulation	ST36	Zeng and Yan [22] (review): ST36 activates α7nAChR-mediated anti-inflammatory signaling; Akoolo et al. [23] (ST36): reduces TNF-α/IL-17 via the sciatic-vagal pathway	Robust reduction in systemic inflammation
Innate Immune & Cytokine Modulation	↓ TNF-α, IL-1β, IL-6, IFN-γ; ↑ IL-10; M1→M2 microglial shift; inhibition of NF-κB, NLRP3 inflammasome	ST36, PC6, LI4, GV20	Ren et al. [24] (GV20): ↓ TRPV4, ↓ TNF-α/IL-6; Zhang et al. [25] (PC6): ↓ NF-κB/COX2; Liu et al. [26] (ST36): ↓ IL-6 and IFN-γ	Consistent suppression of pro-inflammatory cytokines
Neuroimmune–CNS Circuitry	Activation of NTS–LC noradrenergic circuit; limbic/paralimbic modulation; cortical excitability enhancement	ST36, PC6, LI4, GV20	Yu et al. [27] (ST36/ST37): ↑ NTS–LC activation and cognition; Liu et al. [28] (systematic review/meta-analysis): EA/MA ↑ corticospinal excitability	CNS circuit engagement consistently reported
Gut–Brain Axis	Enhanced gastric motility; stabilization of gastric slow waves; improved vagal signaling; modulation of DMV	ST36, PC6	Zhang et al. [19] (ST36): normalizes gastric slow waves; Lu et al. [21] (PC6): inhibits GABAergic input to DMV → ↑ vagal output	Restores GI function via vagal pathways
Pain & Analgesia Mechanisms	Opioid-mediated analgesia; ↓ SP, ↓ CGRP; dorsal horn inhibition; segmental & extra-segmental neuromodulation	LI4, ST36, PC6, LR3	Liu et al. [26] (ST36): ↓ SP/CGRP and microglial activation; Hwang et al. [17]: ST36 and LI4 are core points in pain-control trial patterns with central network engagement described	Strong analgesic and anti-nociceptive effects
Organ Protection & Metabolic Effects	↓ oxidative stress, ↓ apoptosis; improved organ biomarkers (renal, cardiac, brain)	ST36, PC6, GV20	Zeng and Yan [22] (sepsis review): ↓ HMGB1, ↓ oxidative stress markers; Zhang et al. [25] (TBI): ↓ apoptosis and edema	Protective effects across multiple organs

Autonomic Regulation and HRV

Across both human and animal studies, stimulation of ST36 and PC6 emerged as the most consistent modulators of autonomic balance. In healthy volunteers and in experimental stress paradigms, acupuncture or transcutaneous stimulation at ST36 increased high-frequency HRV and reduced low-frequency components, indicating enhanced parasympathetic activity and reduced sympathetic drive. Comparable autonomic effects were observed in clinical populations, including patients with dysmenorrhea and mild hypertension, supporting the translational relevance of these findings.

Preclinical studies reinforced these observations. Electroacupuncture at ST36 increased vagal nerve activity and engaged the cholinergic anti-inflammatory pathway in models of chronic obstructive pulmonary disease and systemic inflammation. Stimulation at PC6 enhanced parasympathetic output and gastric motility through vagovagal reflexes, effects that were markedly attenuated following vagotomy, supporting a causal role for vagal pathways. A structured summary of autonomic and HRV-related effects associated with individual MUAPs is presented in Table 2.

**Table 2:** Frequency of MUAPs Acupoint Appearance Across 73 Included Studies MUAPs: Most Useful Acupuncture Points; CNS: central nervous system; ANS: autonomic nervous system; CAP: cholinergic anti-inflammatory pathway; GI: gastrointestinal; HPA: hypothalamic-pituitary-adrenal; TBI: traumatic brain injury

Acupoint	Number of Studies Using This Point	Primary Mechanistic Domains Represented
ST36 (Zusanli)	46	ANS, CAP, immune modulation, CNS, pain, GI regulation
PC6 (Neiguan)	24	Vagal pathways, gastric motility, pain modulation, CNS regulation
LI4 (Hegu)	19	Analgesia pathways, limbic modulation, corticospinal excitability
LR3 (Taichong)	14	Pain control, limbic regulation, autonomic balance
SP6 (Sanyinjiao)	13	Pain, visceral modulation, HPA-axis effects
GV20 (Baihui)	11	CNS engagement, neuroinflammation, cognitive effects
GV26 (Shuigou)	7	Acute CNS arousal, stroke/TBI models
GB34 (Yanglingquan)	6	Motor excitability, pain, CNS reflex modulation
ST37 & CV6	5	GI and immune modulation; motor pathways
Other points (BL40, SP8, TE5, Taiyang)	1–4	Supplementary roles in pain and segmental modulation

Anti-inflammatory and Neuroimmune Modulation

A second dominant pattern across the evidence base was robust suppression of inflammatory signaling following MUAP-related stimulation. In rodent models of sepsis, burn injury, colitis, arthritis, and pulmonary inflammation, stimulation of ST36 consistently reduced pro-inflammatory cytokines, including tumor necrosis factor alpha (TNF-α), interleukin-6 (IL-6), and interleukin-1β (IL-1β), while downregulating nuclear factor kappa B (NF-κB) and NLRP3 inflammasome signaling. These effects were frequently mediated through α7 nicotinic acetylcholine receptor-dependent mechanisms and vagal-adrenal pathways.

Neuroinflammatory models showed parallel findings. Electroacupuncture at GV20 and GV26 reduced cortical and hippocampal inflammatory markers, attenuated microglial activation, and promoted anti-inflammatory microglial phenotypes in models of ischemic stroke and traumatic brain injury. Collectively, these findings demonstrate that MUAPs exert coordinated immune-modulatory effects across peripheral and central compartments. Key inflammatory and neuroimmune pathways modulated by MUAPs are synthesized in Table 1, with point-specific effects detailed in Table 2.

Central Nervous System and Neural Circuit Engagement

MUAPs also demonstrated convergent effects on central neural circuits involved in autonomic integration, pain modulation, and cognitive regulation. Neuroimaging studies in humans revealed that stimulation of LI4, LR3, ST36, and GV20 modulated activity within limbic-paralimbic-neocortical networks, regions implicated in affective processing, stress regulation, and autonomic control. These effects were distinct from non-acupoint stimulation and correlated with changes in autonomic output and HRV.

In animal models, stimulation of ST36 activated gastric vagal afferents and increased neuronal activity within the nucleus tractus solitarius and locus coeruleus, leading to downstream modulation of hippocampal signaling pathways associated with learning and memory. Meta-analytic evidence further demonstrated that acupuncture at frequently used MUAPs increased corticospinal excitability, with electroacupuncture producing larger effects than manual stimulation. Central nervous system targets and neural circuits associated with MUAP stimulation are summarized in Table 1 and mapped to individual acupoints in Table 2.

Functional and Organ-Specific Outcomes

The physiological effects of MUAPs are translated into measurable functional and clinical outcomes across organ systems. In gastrointestinal models, stimulation of ST36 and PC6 improved gastric motility, normalized slow-wave activity, and reduced visceral hypersensitivity under conditions of stress, injury, and inflammation. In perioperative and pain-related studies, stimulation of LI4 and PC6 reduced analgesic requirements, attenuated inflammatory responses, and improved postoperative recovery and patient-reported outcomes.

Neurological models further demonstrated that MUAP-related stimulation reduced infarct size and neurological deficit scores following ischemic injury, improved cognitive performance in neurodegenerative models, and enhanced neuroprotection in septic and hypoxic brain injury. The translation of MUAPs-related mechanisms into functional and clinical outcome domains is summarized in Table 3.

**Table 3:** MUAPs Clinical Relevance Mapping (Acupoints → Mechanisms → Clinical Domains) LPS: lipopolysaccharide; CLP: cecal ligation and puncture; MUAPs: Most Useful Acupuncture Points; CNS: central nervous system; ANS: autonomic nervous system; CAP: cholinergic anti-inflammatory pathway; GI: gastrointestinal; HPA: hypothalamic-pituitary-adrenal; TBI: traumatic brain injury; HRV: heart rate variability; COPD: chronic obstructive pulmonary disease

Acupoint	Dominant Mechanistic Actions	Key Physiological Pathways	Main Clinical Domains Suggested by Mechanistic Evidence	Example Indications/Study Models
ST36 (Zusanli)	Robust anti-inflammatory and immunomodulatory effects; enhances vagal tone; engages cholinergic anti-inflammatory pathway; stabilizes gastric slow waves; analgesic and organ-protective actions [12,19,22,26,29]	↑ Vagal efferent activity; ↑ HF HRV, ↓ LF/HF; α7nAChR-mediated CAP activation; ↓ TNF-α, IL-1β, IL-6, IFN-γ; ↓ NLRP3, NF-κB; modulation of NTS–DMV and sciatic–vagal circuits; ↓ SP/CGRP [12,14,19,20,23,26,29]	Systemic inflammation/sepsis, GI motility and gut–brain, pain and visceral hypersensitivity, pulmonary and hepatic protection, neuroprotection (stroke, brain injury)	Sepsis (LPS/CLP); COPD; ConA hepatitis; burn-delayed gastric emptying; IBS-D; rectal-distension dyspepsia; inflammatory arthritis; CFA inflammatory pain; APP/PS1 AD model (with ST37) [14,20,23,27,29-33]
PC6 (Neiguan)	Promotes gastric motility; enhances parasympathetic (vagal) output; modulates brainstem vagovagal reflex; contributes to perioperative analgesia and stress reduction [21,34,35]	↑ Parasympathetic discharge; activation of NTS–DMV circuits; inhibition of GABAergic input to DMV; ↓ IL-6; ↑ endogenous opioids (β-endorphin); improved autonomic balance [21,35-37]	Upper GI motility and nausea, perioperative pain and stress, cardiorespiratory and autonomic regulation, neuroprotection after TBI (with GV20/GV26)	Gastric hypomotility; perioperative pain after thoracoscopic surgery/VATS (often with LI4); DMV GABA microinjection model; cardiovagal/autonomic studies [21,35,37]
LI4 (Hegu)	Potent analgesic and neuromodulatory point; modulates ANS; engages limbic/descending pain networks; supports perioperative analgesia [11,35,38]	Segmental and extra-segmental neuromodulation; activation of descending inhibitory circuits; ↑ corticospinal excitability; changes in HF/LF HRV when combined with SP6; ↑ β-endorphin; ↓ IL-6 [11,28,35,38]	Somatic and visceral pain, perioperative pain control, motor cortical excitability modulation, dysmenorrhea and pelvic pain (with SP6)	Primary dysmenorrhea (with SP6); postoperative pain after thoracoscopic surgery (often with PC6); pain RCTs and central/autonomic physiology studies [11,35,38]
SP6 (Sanyinjiao)	Viscerosomatic and pelvic pain modulation; anti-inflammatory analgesia; modulation of pelvic autonomic and sensory pathways [38,39]	↓ TNF-α, IL-1β, PGE2 in pelvic/prostatic tissue; inhibition of P2X7R/NLRP3 inflammasome; modulation of sacral and tibial nerve pathways; changes in HRV with LI4 [38,39]	Gynecologic and pelvic pain, chronic pelvic pain syndrome, dysmenorrhea, visceral pain control	CPPS model (with CV3/CV4/BL35); primary dysmenorrhea (with LI4); inflammatory pelvic pain syndromes [38,39]
LR3 (Taichong)	Distal “core” point for pain and emotional/autonomic modulation; frequently paired with LI4 and ST36 [9,17,40]	Limbic and default-mode network engagement; segmental and extra-segmental pain modulation; contribution to ANS regulation [9,17,40]	Chronic pain, migraine and headache, mood/anxiety-linked pain, visceral pain	RCT-derived point patterns for dysmenorrhea, migraine and osteoarthritis (in combinations with LI4, SP6, ST36) [17,40]
GV20 (Baihui)	Central neuroimmune modulation; neuroprotection; regulation of microglial polarization; improvement in cognition and neuroinflammation [24,25,27,41]	↓ TRPV channels; ↓ TNF-α, IL-6, IL-1β; shift of microglia/macrophages toward M2 phenotype; ↓ NF-κB/COX2 (when combined with multi-point protocols); engagement of cortical–limbic circuits [24,25,27,41]	Ischemic stroke, TBI and brain edema, cognitive impairment and AD, post-sepsis brain injury	MCAO stroke models; TBI models (often with GV26 and/or other points); sepsis-related brain injury models; AD/APP-PS1 models (with ST36/ST37) [24,25,27,41]
GV26 (Shuigou)	Acute CNS arousal and emergency neuroprotection when paired with other MUAPs points [24]	Brainstem and cortical arousal circuits; facilitation of neuroprotective pathways; interaction with NF-κB/COX2 and microglial activity when part of multi-point protocols [24,25]	Acute TBI, coma/emergency resuscitation protocols (preclinical), stroke models	Controlled cortical impact TBI models and acute neuroprotection combinations (commonly paired with GV20 and/or other points) [24]
GB34 (Yanglingquan)	Motor function and corticospinal modulation; segmental neuromodulation for lower limb and trunk [28,42]	Enhanced corticospinal excitability; modulation of descending motor pathways; local–segmental effects at lower limb [42]	Motor recovery after stroke, spasticity and motor control disorders, orthopedic/neurological rehab	Motor-related acupuncture studies in stroke, Parkinson’s disease, and spinal cord injury as part of multi-point motor protocols [42]
ST37 (Shangjuxu)	Distal support point for GI and neuroimmune circuits; amplifies ST36 effects [27]	Vagal afferent activation projecting to NTS; activation of NTS–LC noradrenergic circuit; ↓ hippocampal neuroinflammation [27]	Cognitive impairment, AD models, gut–brain axis modulation	APP/PS1 AD mice (with ST36): improved cognition, ↓ microglial activation, ↓ hippocampal cytokines [27]
CV3/CV4/BL35 cluster	Local pelvic autonomic modulation; anti-inflammatory and anti-hyperalgesic effects in pelvic organs [39]	Parasympathetic sacral outflow; inhibition of P2X7R/NLRP3; ↓ TNF-α, IL-1β, PGE2; improved local blood flow and muscle spasm [39]	Chronic [39]	CFA-induced CPPS models: ↓ inflammatory cytokines, ↓ pain index, improved histology when combined with SP6 [39]

Cross-Point Convergence and Definition of MUAPs

Across all included studies, ST36 was the most frequently studied and mechanistically characterized acupoint, demonstrating reproducible effects on autonomic regulation, inflammatory suppression, neural circuit engagement, and multiorgan functional outcomes. PC6, LI4, LR3, SP6, GV20/GV26, GB34, CV6, TW5, and the auricular vagus zone also showed consistent system-level effects across independent investigations. Network analyses of clinical prescribing patterns confirmed that these points occupy central positions within acupoint networks, exhibiting high connectivity and recurrence across diverse clinical indications. Together, these convergent findings support the identification of MUAPs as a unified, mechanistically coherent cluster of acupoints.

Discussion

This structured narrative review synthesizes mechanistic evidence relevant to a prespecified candidate MUAP cluster rather than establishing a de novo or exhaustive ranking of acupuncture points. Within that narrower aim, the included literature suggests recurrent physiological signals at several candidate points, particularly ST36 and PC6 in the autonomic and vagal domains, ST36, SP6, LI4, GV20, and GV26 in the inflammatory and neuroimmune domains, and LI4, LR3, ST36, and GV20 in central network modulation. These convergent findings support the plausibility of a compact, mechanistically informed candidate framework. They do not, however, validate a standardized clinical protocol or demonstrate superiority over alternative point sets.

A dominant mechanistic theme in the results is the normalization of the autonomic nervous system (ANS), driven most robustly by ST36 and PC6. Multiple studies demonstrate that ST36 stimulation increases vagal activity, suppresses sympathetic overdrive, and restores gastric slow-wave rhythms [43,44]. PC6 similarly enhances parasympathetic output and stabilizes sympathovagal balance [11,36]. This aligns with earlier autonomic models, which show that these points converge on the NTS-DMV circuitry, reinforcing the rationale for their inclusion as core points of MUAPs.

Inflammation modulation also emerged as a defining effect of MUAPs, particularly those at ST36, SP6, and LI11/LI4 combinations. ST36, in particular, reliably activated neuroimmune pathways that down-regulated IL-1β, IL-6, TNF-α, and other pro-inflammatory mediators in models of colitis, periodontitis, and cerebral ischemia [36,43,45]. These findings substantiate the long-standing clinical observation that a subset of acupuncture points exerts broad anti-inflammatory effects, independent of local pathology, supporting their selection as the “most used” points.

A third convergent finding is cortical and limbic network modulation. LI4, LR3, ST36, and GV20 consistently produced deactivation of limbic-paralimbic circuits, increased α/β EEG activity, and improved autonomic-cortical coupling [11]. These effects map onto known neurobiological drivers of anxiety, stress reactivity, chronic pain, and emotional dysregulation, reinforcing the functional logic of a MUAPs cluster designed to address systemic dysregulation rather than narrowly defined disease states.

To contextualize MUAPs within the broader landscape of standardized acupuncture protocols, it is instructive to compare them with widely adopted systems such as NADA, Battlefield Acupuncture (BFA), and the SMART protocol (Stress, Mood, Anxiety, Resilience Treatment).

NADA (auricular detox protocol) uses a fixed set of five auricular points primarily targeting stress regulation, emotional stabilization, and autonomic balance [46,47]. Its global adoption demonstrates the utility of a simple, standardized set-in settings where heterogeneity of trainee background is high. However, NADA is purpose-specific, optimized for behavioral health and addiction recovery, and does not integrate body points or mechanisms related to gastrointestinal, immune, pain, or neuroendocrine regulation [46]. MUAPs expand this logic by incorporating evidence-supported body + auricular points that act through multi-system pathways.

BFA similarly demonstrates the effectiveness of a standardized protocol. BFA relies on auricular points that trigger rapid neuromodulatory analgesia via limbic network modulation [48,49]. Like NADA, BFA is intentionally simple, enabling rapid deployment by clinicians with minimal training. MUAPs share this characteristic simplicity but differ mechanistically: where BFA emphasizes rapid analgesic limbic inhibition, MUAPs emphasize comprehensive autonomic and neuroimmune recalibration, enabling application across chronic multisystem conditions.

The SMART protocol incorporates PC6, LI4, LR3, and auricular vagus points, points that substantially overlap with MUAPs [50,51]. Its success reinforces the broader trend toward autonomic-targeted, evidence-based clusters. MUAPs can be seen as a more complete, mechanistically unified extension of SMART, grounded in a full systematic review and expanded to multiple physiological domains (ANS, HRV, inflammation, cortical networks, gut-brain reflexes).

The evidence synthesized in this review supports the use of MUAPs as a foundational teaching cluster for early trainees. Combining mechanistic clarity with clinical versatility enables rapid competency development while avoiding the cognitive overload of full meridian-based curricula [5]. This is further reinforced by extensive experiential evidence; collectively, the teaching experience of our group exceeds 35 years, during which we have trained more than 1,000 medical students and acupuncture practitioners. Throughout this period, we consistently observed that introducing these 10 points (MUAPs) as a unified foundational cluster greatly accelerates the learning process and improves clinical confidence in early trainees.

Furthermore, our combined clinical experience suggests that MUAPs are particularly effective in treating real-world patients who present with systemic or functional dysregulation, rather than a single, defined disease. These include stress-related autonomic imbalance, insomnia, anxiety, widespread pain and myofascial tension, chronic low-grade inflammation, autoimmune or rheumatologic inflammatory backgrounds, fatigue, subclinical vitality syndromes, and overall reductions in quality-of-life indices. The repeated usefulness of MUAPs across these presentations, observed in both teaching and clinical practice, supports their categorization as a practical, evidence-based core set of points in contemporary biomedical acupuncture.

Importantly, MUAPs align with modern biomedical education by linking each acupoint to identifiable neurophysiological pathways. This addresses well-documented gaps in acupuncture training, such as inconsistency in point selection, poor mechanistic grounding, and insufficient emphasis on evidence-based autonomic and inflammatory physiology [3,4]. The MUAPs cluster also provides a modular framework that can be integrated into individualized treatment planning. It is intentionally foundational rather than exclusive, analogous to how clinicians learn core cardiac or neurological exam maneuvers before developing specialty-specific techniques.

A major strength of MUAPs is the convergence of evidence across multiple study types, including randomized trials, mechanistic models, HRV studies, neuroimaging research, and network analyses, all of which identify the same core set of points as physiological hubs. A second strength is clinical relevance: several MUAPs are already embedded in successful named protocols and have been widely studied across various pathologies, indicating inherent versatility.

Limitations of this review include heterogeneity in stimulation parameters, variation in study quality, and the fact that MUAPs themselves have not yet been clinically validated as a single unified protocol. Additionally, findings from animal models or highly controlled laboratory conditions may not fully generalize to complex human presentations.

Future research should evaluate the candidate MUAP framework prospectively rather than assume its validity from mechanistic synthesis alone. Comparative clinical trials are needed to assess whether MUAP-based clustering improves patient outcomes relative to individualized treatment, standard care, or existing standardized protocols. Mechanistic crossover studies using more homogeneous designs may help clarify the autonomic, neuroimmune, and central signatures associated with specific candidate points or point combinations. Controlled educational studies should examine whether a MUAP-based teaching approach improves learner competence, confidence, retention, or early clinical reasoning compared with broader meridian-based introductory curricula. More systematic comparative work is also needed to determine whether explicit selection criteria, such as recurrence thresholds, network centrality, or mechanistic breadth, can refine or validate the candidate framework.

## Conclusions

MUAPs should be interpreted as a candidate, mechanistically informed acupoint cluster rather than as a validated, standardized protocol. The available literature suggests recurrent autonomic, neuroimmune, and neurophysiological effects across several prespecified candidate points, supporting their relevance as a theoretical framework for biomedical acupuncture teaching and future protocol development. However, the present review does not establish comparative superiority, clinical effectiveness as a unified protocol, or educational effectiveness as a curriculum model. Further prospective clinical, mechanistic, and educational research is needed before stronger claims can be made.

## Appendices

Supplemental material 1

**Table 4:** Detailed Study Characteristics

Author(s)	Year	Country/Setting	Study Type	Population/Model	Comparator	Condition Modeled	Intervention Type (Acupuncture Type)	Sessions	Frequency (Hz), Intensity (mA), Waveform	Duration per Session	Acupoints Used	MUAPs Present	ANS/HRV Outcomes	Vagus/Cholinergic Evidence	Inflammation Markers	CNS/Neuroimmune Mechanisms	Pain Outcomes	Other Outcomes	Mechanistic Insight	Primary Outcome	Secondary Outcome	Effect Direction	Conclusions
Allais et al. [40]	2002	Italy	RCT	Human	Active drug comparator (flunarizine)	Migraine without aura	Manual acupuncture (De Qi–elicited; manipulation described)	Weekly sessions for the first 2 months, then monthly sessions for the next 4 months	Not Reported	20 minutes	LR3; SP6; ST36; CV12; LI4; PC6; GB20; GB14; EX-HN5; GV20	LI4; PC6; ST36; SP6; LR3; GV20	Not reported	Not reported	Not reported	Not reported	Not Reported	Not Reported	Not Reported	Decrease in frequency of attacks was the main efficacy parameter; acupuncture was significantly lower than flunarizine after 2 months: T1A 2.95 ± 0.39 vs T1F 4.10 ± 0.42 (95% CI 0.02–2.28) and T2A 2.30 ± 0.20 vs T2F 2.93 ± 0.24 (95% CI 0.02–1.24); unpaired t test for both comparisons, p < 0.05.	Pain intensity was significantly reduced only by acupuncture treatment. Analgesic consumption was significantly lower in acupuncture vs flunarizine at T1 (T1A 5.13 ± 0.46 vs T1F 6.70 ± 0.56; 95% CI 0.14–3.00; unpaired t test, p < 0.05). Side effects were significantly lower in acupuncture vs flunarizine (10/77 vs 29/73; χ² = 7.22, 1 df; p = 0.007).	Frequency of attacks decreased (↓); Pain intensity decreased (↓) (Group A only); Analgesic doses decreased (↓).	Acupuncture was effective for migraine prophylaxis, showing superior early efficacy and tolerability compared with flunarizine.
Hübscher et al. [34]	2007	Germany	RCT-double blind	Human	Sham laser stimulation (inactive output)	Healthy volunteers	Laser acupuncture	Single intervention session	Red light (685 nm) and infrared light (880–950 nm), continuous wave, output power 30–40 mW per laser needle	20 minutes	PC6	PC6	Laser: ln LF/HF (pre, stim-1, stim-2, post): 0.51 (0.75), 0.32 (0.86), 0.51 (0.81), 0.30 (0.94). Placebo: ln LF/HF (pre, stim-1, stim-2, post): 0.47 (0.73), 0.44 (0.70), 0.47 (0.52), 0.50 (0.46).	Not reported	Not reported	Not reported	Not Reported	Not Reported	Not Reported	Repeated-measures MANOVA showed no significant main effects of time (F = 1.29, p = 0.27) or group (F = 1.67, p = 0.16), and no significant time × group interaction (F = 0.95, p = 0.54).	Not reported	Not applicable	Laser needle acupuncture at PC6 did not significantly alter heart rate variability.
Streitberger et al. [11]	2008	Germany	RCT-single blind	Human	Sham acupuncture at non-acupoint	Healthy volunteers	Manual acupuncture	Two sessions on separate days	Not reported	15 seconds	LI4	LI4	During stimulation, LF/HF increased initially (sympathetic activation). HF increased in the minute after stimulation. HF (VA vs PA, first minute post-stimulation): 0.20 ± 0.18 vs −0.01 ± 0.57 (p ≤ 0.05). LF/HF (first minute of stimulation): 0.52 ± 0.37 vs −0.14 ± 0.25 (p ≤ 0.02). HR decreased in the minute after HF increase (p ≤ 0.041).	Not reported (general discussion of enhanced vagal and suppressed sympathetic activity).	Not reported	Needle stimulation in VA increased α1-frequency significantly; α1/β ratio shifted toward α1 across electrodes; VA increased occipital α1 power; negative linear correlation between β-band EEG and HRV parameters (LF and HF) during VA.	Pain was significantly more intense during stimulation in VA compared to PA. Stimulation mean ± SEM: 2.40 ± 0.54 versus 0.78 ± 0.16, p = 0.001. De qi sensation was felt. The VAS score showed a significant correlation with the LF and HF band.	Not Reported	Not Reported	Not reported (study evaluated specific effects on central and vegetative nervous system activity).	EEG frequency changes; HR changes; vegetative side effects; pain assessment.	LF/HF ratio (during stimulation) ↑; HF power (after stimulation) ↑; Heart rate (HR) ↓; α1-frequency (occipital area) ↑.	Manual acupuncture at LI4 induced central and autonomic nervous system modulation consistent with a relaxation effect.
Wu et al. [37]	2009	Taiwan	RCT- double blind	Human	Sham laser stimulation (inactive output)	Circadian rhythm disruption (night-shift work)	Laser acupuncture	Single session	Wavelength 830 nm; frequency 10 Hz; output power 60 mW; energy density 9.7 J/cm²; duty cycle 50%	10 minutes	PC6	PC6	Compared with placebo, laser improved HRV: HF (p ≤ 0.001), LF (p ≤ 0.001), LF/HF (p ≤ 0.02). After treatment: HF% 55.33 ± 16.15 vs 35.60 ± 16.26 (p = 0.002); LF% 44.67 ± 16.15 vs 64.40 ± 16.26 (p = 0.002); LF/HF 1.02 ± 0.76 vs 2.35 ± 1.35 (p = 0.003).	Laser acupuncture at PC6 increased vagal activity and suppressed cardiac sympathetic activity (HF 0.15–0.40 Hz as PNS proxy; LF 0.04–0.15 Hz as mixed SNS/PNS proxy).	Not reported	Heart rhythm is controlled by ANS centered in brain; prior work suggests laser acupuncture activates cortical areas via point stimulation, modulating neuronal networks; mechanical impulses via connective tissue may activate signal transduction pathways and alter ANS modulation.	Not reported.	Not Reported	Not Reported	Not reported (study evaluated ANS impact; primary effects reported included increased HF HRV (p < 0.001), decreased LF HRV (p < 0.001), and decreased LF:HF ratio (p = 0.02)).	Blood pressure (SBP, DBP) and HR; HR improved after treatment in treated group (p < 0.05); SBP, DBP, and HR showed no significant between-group differences after treatment or rest (p > 0.05).	HF HRV ↑; LF HRV ↓; LF:HF ratio ↓; HR ↓.	Laser acupuncture at PC6 enhanced vagal activity and suppressed cardiac sympathetic tone, indicating improved autonomic balance.
Fang et al. [9]	2009	China and USA	fMRI study	Human	Sham acupuncture at non-acupoint (dorsum of foot)	Healthy volunteers (central nervous system response)	Manual acupuncture	Single session	Not Reported	6 minutes	LR3; LV2; ST44	LR3	Not reported	Limbic–paralimbic–neocortical circuitry implicated in regulation/integration of autonomic, endocrine, immunologic, and sensorimotor functions.	Not reported	Acupuncture deactivated limbic-paralimbic-neocortical system (medial prefrontal cortex, temporal lobe including amygdala/hippocampus/parahippocampus, posterior medial cortex including precuneus/posterior cingulate) and activated sensorimotor cortices, thalamus, and occasionally insula/anterior middle cingulate; effects mediated via CNS.	Acupuncture may mediate its antipain, antianxiety, and other therapeutic effects via this intrinsic neural circuit that plays a central role in the affective and cognitive dimensions of pain. Deactivation of the amygdala and hippocampus was found to correlate with the elevation of pain threshold in the subjects. The release of opioid peptides in the central nervous system is the primary mechanism implicated in the analgesic effect of acupuncture.	Not Reported	Not Reported	Primary outcome focused on central effects (BOLD signal changes) and psychophysical responses; differences between acupoints and sham were limited (e.g., PCC/BA31 deactivation with sham but not LV2/ST44; left temporal pole activation for LV2 but deactivation for sham; p ≤ 0.005 uncorrected).	Psychophysical response (sensory experience/deqi): aching, soreness, numbness, distension.	Limbic–paralimbic–neocortical system deactivation (↓); sensorimotor cortices activation (↑); thalamus activation (↑); occasional paralimbic activation (↑) (insula, anterior middle cingulate).	Acupuncture produced widespread limbic and paralimbic deactivation with sensorimotor activation, supporting central neuromodulatory effects while acupoint specificity remains uncertain.
Sakatani et al. [52]	2010	Japan	Physiological monitoring	Human	Sham acupuncture near LI4 (non-acupoint)	Healthy adults	Manual acupuncture (bidirectional needle rotation)	Single session	Not Reported	2.5 minutes	LI4	LI4	Real acupuncture at LI4 decreased HR (p = 0.0409) and ln LF/HF (p = 0.0044), and increased ln HF (p = 0.0087). ln HF: 5.16 ± 0.18 to 5.57 ± 0.19. ln LF/HF: 1.12 ± 0.05 to 1.00 ± 0.04.	Not reported	Not reported	Prefrontal cortex oxygenation (Oxy-Hb) changed bidirectionally in bilateral PFC; with l-LI4, change in Ln LF correlated with Oxy-Hb change in r-APFC (p = 0.009; r = 0.668) and Ln HF correlated with Oxy-Hb change in r-LPFC (p = 0.0166; r = 0.648).	Not reported.	HPA/stress axis effects	The prefrontal cortex plays an important role in mediating behavioral and somatic responses to stress via projections to autonomic centers.	Ln LF/HF ratio decreased significantly with real acupuncture at r-LI4 (p = 0.0044).	HR and Oxy-Hb changes in bilateral PFC; r-LI4 decreased HR (p = 0.0409) and increased Ln HF (p = 0.0087).	HR ↓; LF/HF ratio ↓; HF power ↑; Oxy-Hb change: increase and/or decrease.	Real acupuncture shifted autonomic balance toward parasympathetic dominance, reflected by increased HF and reduced LF/HF ratio.
Kim et al. [38]	2011	South Korea and Australia	Crossover and patient-blinded procedure	Human	Sham acupuncture using superficial needle insertion	Primary dysmenorrhea	Manual acupuncture	Two-session crossover with a one-month washout period	Not reported	15 minutes	LI4; SP6	LI4; SP6	Real acupuncture decreased LF/HF and increased HF. HF (ms²): 562.83 ± 96.33 to 806.73 ± 119.12 (p < 0.001). LF/HF: 1.60 ± 0.21 to 1.05 ± 0.12 (p = 0.001). LF (ms²): 554.59 ± 61.31 to 694.07 ± 111.32 (p = 0.171).	Not reported	ta-VNS/VNS inhibited TNF-α, IL-1β, and IL-6; TEAS at ST36 lowered serum TNF-α but did not significantly change IL-1β or IL-6.	Not reported	Not Reported	Not Reported	Not Reported	HF power increased significantly (p < 0.001) and LF/HF ratio decreased significantly (p = 0.001) following stimulation of Hegu and Sanyinjiao points.	Menstrual pain VAS decreased with both real and sham acupuncture (data not shown); sham acupuncture increased HF power post-stimulation (p = 0.004).	HF power (RA) ↑; LF/HF ratio (RA) ↓; menstrual pain VAS ↓.	Manual acupuncture at LI4 and SP6 alleviated dysmenorrhea symptoms through autonomic nervous system involvement.
Zhao et al. [14]	2012	China	Mechanistic animal study	Animal	Disease model control (endotoxemia, LPS)	Lipopolysaccharide-induced systemic inflammation (endotoxemia)	Transcutaneous auricular vagus nerve stimulation (taVNS) and transcutaneous electrical acupoint stimulation (TEAS)	Not reported	Constant electrical current, 1 mA, 10 Hz, pulse width 1 ms	20 minutes	ST36; Auricular concha	ST36	Not reported	ta-VNS may suppress LPS-induced inflammation via α7nAChR-mediated cholinergic anti-inflammatory pathway.	Not reported	ta-VNS: afferent vagal signals transmit to NTS; TEAS at ST36: somatic fiber endings send signals via somatic sensory fibers to spinal cord, then via secondary neurons to NTS for processing.	Not Reported	Not Reported	Not Reported	Cytokine suppression: electrical VNS and ta-VNS inhibited LPS-induced TNF-α, IL-1β, and IL-6 (n = 12; p < 0.01 and p < 0.05, respectively).	NF-κB p65 suppression: electrical VNS and ta-VNS inhibited LPS-induced NF-κB p65 (n = 10, p < 0.01); ST36 did not significantly affect NF-κB p65.	Cytokine concentrations ↓; NF-κB p65 expression ↓.	ta-VNS and stimulation at ST36 modulated immune responses by activating the cholinergic anti-inflammatory pathway and suppressing pro-inflammatory signaling.
Witt et al. [36]	2012	Germany	RCT- single blind	Human	Sham acupuncture	Healthy volunteers	Manual acupuncture (verum and non-stimulated variants)	Single session with sequential stimulated and non-stimulated phases	Not reported	30 minutes	ST36; PC6	PC6; ST36	RMSSD higher with stimulated vs non-stimulated verum acupuncture (p < 0.001): 3.8 ± 0.1 vs. 3.7 ± 0.1 (difference 0.2 [0.1–0.2]). Trend toward higher HF with stimulation. No meaningful change in sympathetic indices (skin conductance, stroke volume, peripheral vascular resistance).	Not reported (focus on autonomic modulation/cardiac vagal tone).	Not reported	Neuroimaging suggests acupuncture can activate hypothalamus and periaqueductal gray, implicating higher centers of autonomic control.	Not Reported	Gut–brain axis findings	Acupuncture affects gastric myoelectrical activity, altering the proportion of normogastria and bradygastria.	Primary outcome was percentage of regular gastric slow waves (normogastria); no significant difference between verum and sham acupuncture (VA 31.7 ± 2.5 vs SA 32.8 ± 2.4, p = 0.757).	sVA vs nsVA: normogastria lower (p = 0.005), bradygastria higher (p = 0.003), power ratio higher (p < 0.001), SBP lower (p = 0.039), RMSSD higher (p < 0.001).	Normogastria ↓ (during sVA); bradygastria ↑ (during sVA); power ratio ↑ (during sVA); systolic blood pressure ↓ (during sVA); RMSSD ↑ (during sVA).	Acupuncture at ST36 and PC6 altered gastric myoelectrical activity and cardiac autonomic function, highlighting interactions between gastrointestinal and cardiovascular regulation.
Gao et al. [15]	2012	China and Austria	mechanistic animal study	Animal	Not applicable (acupoint-to-acupoint comparison without sham control)	Healthy anesthetized animals	Manual acupuncture	Single session	Not reported	30 seconds	Auricular Heart; PC6; ST36; CV12	PC6; ST36	HR unchanged across stimulations. Total HRV increased during auricular stimulation (“heart” point). LF/HF decreased during ST36 stimulation (p = 0.03).	Not reported (autonomic control and BP regulation discussed).	Not reported	ST36-related mechanism linked to BP regulation; auricular acupuncture regulates cardiovascular function via cardiac-related and depressor neurons in NTS similar to baroreceptor reflex; RR-interval regulation influenced by hypothalamus and lower brainstem vagal cardiovascular center.	Not Reported	The mechanism related to ST36 stimulation is based on intensification of blood pressure regulation. Auricular acupuncture regulates cardiovascular function by activating cardiac-related and depressor neurons in the nucleus tractus solitarius in a manner similar to the baroreceptor reflex. RR-interval regulation is influenced by the hypothalamus and the vagal cardiovascular center in the lower brainstem.	Acupuncture affects gastric myoelectrical activity, altering the proportion of normogastria and bradygastria.	Not reported (primary goal was to register/analyze HR and HRV); HRV total increased significantly (p = 0.025) only after manual auricular acupuncture at Heart point.	Mean HR changes (not significant); LF/HF ratio changes.	HRV total (auricular Heart) ↑; LF/HF ratio (ST36) ↓.	Auricular acupuncture significantly increased total HRV, while ST36 selectively reduced LF/HF ratio through blood pressure regulatory mechanisms.
Lee et al. [53]	2012	Republic of Korea	mechanistic animal study	Animal	Disease model control (CFA-induced inflammatory pain)	CFA-induced inflammatory hyperalgesia	Electroacupuncture	Once daily for 2 consecutive days after CFA injection	2 Hz, 1 mA, electrical pulse stimulation	20 minutes	ST36; SP6	ST36; SP6	Not reported	Not reported	Not reported	Spinal cord dorsal horn phosphoproteome/nociceptive signaling: persistent noxious stimuli increase excitability of secondary neurons and lower pain threshold; phosphorylation profiling informs altered patterns of proteins involved in EA analgesia in SCDH.	The phosphorylation changes of nociceptive signaling proteins in the spinal cord dorsal horn are important in creating exaggerated pain following peripheral inflammation. EA may exert an analgesic effect by inhibiting Rho GTPase-regulated spine remodeling through the dephosphorylation of GDI1. Hsp90 is involved in microglial TLR4-mediated pain enhancement in rat spinal cord dorsal horn. Eight proteins were differentially phosphorylated following EA treatment in the inflammatory pain model. Aldolase C, nascent polypeptide-associated complex α, stress-induced phosphoprotein 1 and heat shock protein 90 were identified as phosphoproteins whose expression was increased, whereas GDP dissociation inhibitor 1, thiamine triphosphatase, phosphoglycerate kinase 1 and 14-3-3 γ were phosphoproteins whose expression was decreased.	Not Reported	Not Reported	Paw withdrawal latency (PWL) improved significantly after EA vs CFA inflammatory pain group (p < 0.001; CFA PWL ≈ 4.2 s; CFA+EA PWL ≈ 6.8 s).	Differential phosphorylation: eight proteins differed after EA; STI1 and Hsp90 phosphorylation decreased in pain model vs control and were restored by EA; EA decreased phosphorylation vs inflammatory pain group for several targets (e.g., GDI1/PGK1/14-3-3γ details as reported).	Paw withdrawal latency (PWL) ↑; Aldolase C, NACA, STI1, Hsp90 phosphorylation (vs CFA) ↑; GDI1, thiamine triphosphatase, PGK1, 14-3-3γ phosphorylation (vs CFA) ↓.	Electroacupuncture analgesia was associated with regulation of intracellular protein phosphorylation pathways involved in pain processing.
Liu et al. [20]	2012	China and USA	A two-session study (AP and sham-AP, AP and no-AP, or sham-AP and no-AP) involving human volunteers.	Human	Sham acupuncture at non-acupoint (lateral to ST36)	Rectal distension–induced visceral hypersensitivity	Manual acupuncture	Two sessions per experiment across three experiments	Not reported	30 minutes	ST36	ST36	During rectal distension, acupuncture increased vagal activity (HF) and reduced sympathetic activity (LF). HF increased from 0.43 ± 0.28 to 0.59 ± 0.19 (p ≤ 0.01). LF decreased from 0.52 ± 0.27 to 0.41 ± 0.19 (p ≤ 0.05).	AP at ST36 may improve RD-induced abdominal symptoms and impaired gastric slow waves via a vagal efferent pathway.	Not reported	AP analgesia described as mediated by endogenous opioid pathway; EA at ST36 effects abolished by naloxone in prior canine study, supporting central opioid pathway; impaired gastric slow waves attributed to intrinsic autonomic functions.	The analgesic effect of AP has been reported to be mediated by an endogenous opioid pathway.	Gut–brain axis findings	Rectal distension induces dyspepsia symptoms, inhibits gastric accommodation, and delays gastric emptying via an inhibitory rectogastric reflex; symptom improvement is believed to be mediated through somatosensory vagal pathways	AP (not sham-AP/no-AP) reduced upper and lower abdominal symptoms (p ≤ 0.05); lower abdominal score 8.09 ± 0.23 → 5.75 ± 0.21 (p ≤ 0.01) and upper abdominal score 3.01 ± 0.59 → 1.50 ± 0.42 (p ≤ 0.01).	RD reduced % normal gastric slow waves (p ≤ 0.05); AP improved gastric slow waves vs sham/no-AP (p ≤ 0.05); during second 30-min RD with AP, normal slow waves 74.0% ± 11.9% (no change vs prior period, p ≥ 0.05) and higher than sham session (p ≤ 0.01); AP increased HF (p ≤ 0.01).	Upper abdominal symptoms ↓; lower abdominal symptoms ↓; percentage of normal gastric slow waves ↑; vagal activity (HF) ↑; sympathetic activity (LF) ↓.	Abdominal acupuncture at ST36 improved gastric slow waves and abdominal symptoms via vagal pathway activation.
Wang et al. [54]	2013	China and Austria	experimental animal study	Animal	Pharmacological blockade (propranolol; atropine) and vagotomy	Healthy volunteers	Manual acupuncture	Not reported	20 minutes	PC6	PC6	Autonomic blockade comparisons reported (atropine/propranolol; vagotomy) with HRV values: Control 37.27 ± 16.20; 45.02 ± 25.32; 0.91 ± 0.32. After atropine/propranolol: 14.44 ± 5.75; 22.97 ± 17.45; 1.57 ± 1.05. After vagotomy: 1.86 ± 0.86; 0.28 ± 0.16; 6.38 ± 1.46.	Not reported	Not reported	Acupuncture effects on HRV rely on full CNS involvement; hypothalamus, rostral ventrolateral medulla, arcuate nucleus, and ventrolateral periaqueductal gray implicated in attenuation of sympathoexcitatory cardiovascular reflexes; long-loop pathway contributes to sustained reduction in sympathetic premotor outflow.	Not Reported	Not Reported	Not Reported	After autonomic blockade, LF and HF increased but only HF increased significantly (p < 0.01), producing significant LF/HF change (p < 0.01); after cervical vagotomy, LF/HF and LF increased significantly (both p < 0.01) with no significant HF change.	Not reported	After drug block: HF ↑ and LF/HF ↓. After vagotomy: LF ↑ and LF/HF ↑.	Acupuncture modulated heart rate variability even after pharmacologic autonomic blockade, indicating combined central and autonomic mechanisms.	
Kaneko et al. [55]	2013	Japan	Randomized Crossover Trial	Human	No-stimulation control	Reduced gastrointestinal motility (ethanol-induced)	Manual acupuncture	Crossover sessions with at least 7 days between trials	Not reported	15 minutes	ST36; LR3	ST36; LR3	ST36: VLF +479.4 ± 1185.6 (p = 0.045); LF +123.9 ± 217.2 (p = 0.006); HF +78.9 ± 197.6 (p = 0.048); LF/HF +71.5 ± 171.1 (p = 0.039). LR3: HF decreased −18.2 ± 35.8 (p = 0.014).	Not reported	Not reported	Not reported	Not Reported	Gut–brain axis findings	ST36 has beneficial effects on gastrointestinal symptoms, potentially mediated by altered intestinal blood flow via reduced sympathetic activity and enhanced vagal activity.	Superior mesenteric artery blood flow volume increased after ST36 stimulation (0% to 14.1% ± 23.4%, p = 0.007).	SBP decreased (p = 0.037); VLF, LF, HF, and LF/HF increased significantly.	SMA BFV ↑; SBP ↓; VLF ↑↑; LF ↑↑; HF ↑↑; LF/HF ↑↑.	Increased vagal activity following ST36 stimulation enhanced superior mesenteric artery blood flow.
Li et al. [56]	2013	China	mechanistic animal study	Animal	Pharmacological blockade (atropine)	Chronic functional constipation	Electroacupuncture (auricular and somatic)	Single session	2 Hz, 1 mA; waveform not reported	15 minutes	Auricular ST; Auricular SI; ST36	ST36	No significant differences in LF, HF, or LF/HF versus control in auricular or somatic acupuncture groups.	Auricular acupuncture effects on GI motility are regulated by the vagus and cholinergic pathways; atropine blockade suggests acetylcholine–muscarinic receptor involvement.	Not reported	Vagovagal reflex neurocircuit: NTS as primary brainstem site receiving vagal afferents; DMV as initiation site of efferent output.	Not Reported	Gut–brain axis findings	Auricular and somatic acupuncture enhance gastrointestinal transit; vagal pathways involved in GI motility may be distinct from cardiac vagal pathways.	GI transit rate increased with AA and SA vs control (70.72 ± 10.28 vs 56.49 ± 12.09, p = 0.011; 73.38 ± 10.28 vs 56.49 ± 12.09, p = 0.003).	Heart rate variability (HRV).	GI transit rate ↑.	Auricular acupuncture regulated gastrointestinal motility through auricular branch vagal nerve mechanisms comparable to sham stimulation.
Chen et al. [57]	2013	Taiwan	RCT-single blind	Human	Sham electroacupuncture at non-acupoint	Cerebral ischemia–reperfusion injury (middle cerebral artery occlusion)	Electroacupuncture	Eight weekly sessions	15 Hz, 5–10 mA; pulse width 20 μs	20 minutes	ST36; ST37; ST25; ST28; CV4; CV6	ST36; CV6	With EA over time: nHFP increased (week 1: 26.79; week 4: 32.28; week 8: 37.60; p < 0.05). nLFP decreased (week 1: 30.81; week 3: 25.98; week 8: 24.84; p < 0.05). LFP/HFP decreased at week 8: 0.825 (0.57–1.10) (p < 0.05).	Not reported	EA reduced inflammatory cytokines TNF-α, IL-1β, and IL-6 (e.g., TNF-α ~220 to ~105 pg/mL; IL-1β ~60 to ~45 pg/mL; IL-6 ~290 to ~175 pg/mL).	CNS effects described in defecation reflex regulation: sympathetic signals to spinal cord; parasympathetic signals increase peristalsis via sacral reflex.	Not Reported	Gut–brain axis findings	Electroacupuncture improves gastrointestinal peristalsis by reducing sympathetic activity and enhancing parasympathetic tone.	Defecation “total effect” after eight weeks: electroacupuncture 93.4% vs non-electroacupuncture 64.3%; significant group difference after four weeks (p = 0.037).	HRV power spectral density changes (nLFP, nHFP, LFP/HFP ratio).	Defecation total effect ↑; nHFP ↑; nLFP ↓; LFP/HFP ratio ↓.	Electroacupuncture enhanced parasympathetic activation and improved constipation outcomes following prolonged treatment.
Lan et al. [43]	2013	China	mechanistic animal study	Animal	Ischemia control and sham operation control	Burn-induced delayed gastric emptying	Electroacupuncture	Single session post-operation with 24-hour follow-up	1 Hz or 20 Hz; intensity at muscle twitch threshold (~0.01 mA)	Not reported	LI11; ST36	ST36	Not reported	Not reported	EA attenuated burn-induced plasma IL-6 increase at 6 h and 24 h post-burn (e.g., 6 h: 258.0 ± 48.9 vs 386.6 ± 29 pg/mL; 24 h: 195.9 ± 20.2 vs 275.6 ± 10.4 pg/mL).	Focal cerebral I/R model: mechanism via TLR4/NF-κB pathway; EA reduced inflammatory infiltration in infarct areas, inhibited NF-κB nuclear translocation, and reduced TLR4/NF-κB p65 expression and IκB phosphorylation compared with IC group.	Not reported.	Not Reported	Not Reported	Neurological function improved at 24 h (EA 1.38 ± 0.52 vs IC 2.13 ± 0.83, p < 0.05) and infarct volume reduced (EA ~16.7% vs IC ~34%, p < 0.05).	Inflammation outcomes: reduced cytokines (TNF-α, IL-1β, IL-6) and suppressed TLR4/NF-κB signaling.	Neurological deficits score ↓; cerebral infarct volume ↓; TNF-α/IL-1β/IL-6 ↓; NF-κB nuclear translocation ↓.	Electroacupuncture exerted neuroprotection in ischemic stroke by inhibiting TLR4/NF-κB–mediated inflammatory pathways.
Song et al. [29]	2013	USA and China	mechanistic animal study	Animal		Healthy subjects and functional dyspepsia	Electroacupuncture	Acute stimulation at 6 hours or 24 hours post-burn injury	25 Hz, 4 mA; train on-time 2 s, off-time 3 s	30 minutes	ST36	ST36	EA increased vagal activity (HF) post-burn versus sham: 6 h HF 0.78 ± 0.06 vs 0.29 ± 0.14 (p = 0.004); 24 h HF 0.94 ± 0.15 vs 0.48 ± 0.20 (p = 0.03).	Vagotomy blocked EA acceleration of gastric emptying in burn model, supporting a vagal mechanism; IL-6 implicated.	Not reported	EA at ST36 proposed to activate NTS and stimulate DMV, enhancing gastric motility; ST36 increased c-Fos–positive cells in caudal NTS and DMV in cited work.	Not Reported	Gut–brain axis findings	Gastric emptying correlates positively with vagal activity and negatively with IL-6, indicating vagal mediation of electroacupuncture effects on gastric motility after burn injury.	Gastric emptying improved 6 h and 24 h post-burn with EA (p < 0.001); 6 h GE 74.7 ± 3.1% (EA) vs 51.4 ± 4.0% (sham), p = 0.0003.	Burn model: EA improved gastric dysrhythmia; normal slow waves increased at 6 and 24 h (p = 0.02); GE correlated positively with HF at 24 h (R = 0.5, p = 0.02) and negatively with plasma IL-6 at 24 h (R = −0.6, p = 0.0095).	Gastric emptying (GE) ↑; normal slow waves (N%) ↑; vagal activity (HF) ↑; plasma IL-6 ↓.	Electroacupuncture at ST36 improved gastric dysrhythmia and accelerated gastric emptying through vagal and cytokine-mediated mechanisms.
Chung et al. [58]	2014	South Korea, Taiwan, USA, Germany, China, and Japan	SLR and MA	Human	Sham acupuncture or no-treatment control	Acute myocardial ischemia–reperfusion injury and postoperative cognitive dysfunction	Needle acupuncture with or without electrical stimulation	Multiple sessions reported; single-session studies were excluded	Not reported	Not reported	PC6; SP4; HT7; LI4; SP6	LI4; PC6; ST36; SP6	Meta-analysis: non-healthy LF/HF overall WMD −0.50 (95% CI −0.56 to −0.44), p < 0.00001. Healthy LF norm WMD −0.30 (95% CI −0.44 to −0.15), p < 0.0001. ST36 effects on HF in healthy subjects reported (Z = 3.00; p = 0.003).	Not reported	IL-6 and TNF-α measured in serum and hippocampal tissue; both remained elevated post-reperfusion, with highest TNF-α in AMIR group across reperfusion days.	Not reported	Not Reported	Not Reported	Not Reported	Meta-analytic primary findings: acupuncture reduced LF and LF/HF in nonhealthy subjects and reduced LF norm in healthy subjects (nonhealthy LF/HF overall Z = 15.82, p < 0.00001; healthy LF norm overall Z = 4.03, p < 0.0001).	Meta-analysis: overall effects favored sham/control (not acupuncture) for HF in nonhealthy and HF norm in healthy subjects (nonhealthy HF Z = 5.25, p < 0.00001; healthy HF norm Z = 5.00, p < 0.00001).	LF ↓ and LF/HF ratio ↓ (nonhealthy); LF norm ↓ (healthy).	Evidence from systematic synthesis indicates acupuncture preferentially modulates low-frequency HRV components rather than HF components.
Yuan et al.[59]	2014	China	mechanistic animal study	Animal	Sham operation control	Startle response model	Electroacupuncture	Single session	5 Hz, 3 mA; pulse duration 100 ms, interval 400 ms	30 minutes	GV20; ST36	ST36; GV20	LF/HF was higher in AMIR than other groups at days 1 and 3 (p < 0.05). Day 3 LF/HF: AMIR 2.36 ± 0.85 vs EA 1.64 ± 0.14.	CAP described as α7 AChR-dependent; EA hypothesized to activate CAP via vagus nerve (acetylcholine as major transmitter).	Not reported	Hippocampal CA1 pathology improved; EA suppressed microglial activation (Iba-1 positive cells reduced vs AMIR) and inhibited neuronal apoptosis (TUNEL-positive cells reduced) across reperfusion timepoints.	Not Reported	Not Reported	Not Reported	Survival: AMIR 40% (8/20) vs sham 100% (20/20), p < 0.05; EA 75% (15/20) higher than AMIR, p < 0.05; EA improved working and reference memory errors (p < 0.05).	Oxidative stress (MDA, SOD); histopathology (hippocampal CA1 morphology); inflammation (IL-6, TNF-α).	Survival rate ↑; microglial activation ↓; neuronic apoptosis ↓; MDA ↓; SOD activity ↑; working memory errors ↓; reference memory errors ↓.	Electroacupuncture attenuated autonomic imbalance, oxidative stress, neuroinflammation, and neuronal apoptosis following acute myocardial ischemia-reperfusion.
Villas-Boas et al. [60]	2015	Brazil	mechanistic animal study	Animal	Non-acupoint needle insertion control	Cold stress–induced gastric dysmotility	Needle acupuncture	Single session	Not Reported	20 minutes	GV1; HT7; BL52; GV20	GV20	Acupuncture shifted sympathovagal balance toward parasympathetic modulation and reduced startle-induced LF/HF increase. LF differed vs control (p = 0.016); HF differed vs control (p = 0.008); LF/HF lower vs control (p = 0.002).	Not reported	Not reported	Acupuncture conceptualized as sensory stimulation engaging afferent fibers; signals to spinal cord and higher CNS centers trigger neurophysiological events producing therapeutic effects.	Not Reported	HPA/stress axis effects	Acupuncture reduces startle-induced cortisol elevation via activation of autonomic centers and the hypothalamic–pituitary–adrenal axis.	ACUP group had lower LF/HF than control (p = 0.002); ACUP differed from control at 30 min post-startle (p < 0.01).	No behavioral changes; HR lower in acupuncture vs NP (p = 0.014); startle response latency/duration/distance not different (p = 0.1728, 0.9754, 0.1845).	LF/HF ratio ↓; cortisol (30 min after startle) ↓; LF power ↓; HF power ↑.	Single-session acupuncture reduced startle-induced sympathetic activation and cortisol responses without altering behavior.
Zhang et al. [19]	2015	China and USA	A four-session study (control, cold stress, TEA, and sham TEA)	Human	Sham transcutaneous electrical acustimulation at non-acupoint	Rheumatoid arthritis	Transcutaneous electroacupuncture (TEA; needle-less)	Four sessions on separate days in randomized order	25 Hz; intensity at maximal tolerable level; train on-time 2 s, off-time 3 s; pulse width 0.5 ms	30 minutes	ST36	ST36	Cold stress increased sympathetic tone and reduced vagal activity; TEA at ST36 increased vagal activity and improved balance. TEA suppressed meal-related LF/HF increase (0.77 ± 0.19 vs 1.35 ± 0.24; p < 0.05).	Vagal mechanism proposed for ST36 effects on gastric motility: somatic afferents → NTS → DMV → increased vagal efferent output to viscera.	Laser acupuncture reduced inflammatory markers in RA: CRP, IL-6, and ESR decreased (e.g., CRP 54.40 ± 6.40 to 15.470 ± 2.063 mg/L; IL-6 103.143 ± 9.984 to 67.012 ± 6.932; p values as reported).	Afferent signal conveyed to NTS and activates DMV; broader CNS engagement associated with reduced sympathetic outflow.	Not Reported	Gut–brain axis findings	Stress alters gastrointestinal motility; transcutaneous electroacupuncture improves stress-induced gastric slow-wave impairment via autonomic mechanisms.	TEA at ST36 (not sham) normalized gastric slow waves under stress (p = 0.03 vs stress; p = 0.44 vs control); primary outcome was restoration of percentage of normal slow waves.	Cold stress: impaired normal slow waves (88.6% ± 3.8% control vs 71.0% ± 6.9% stress, p < 0.01), increased bradygastria/tachygastria and instability coefficient; TEA (not sham) abolished instability effects.	Percentage of normal slow waves (TEA) ↑; bradygastria (TEA) ↓; tachygastria (TEA) ↓; instability coefficient ↓; vagal activity (HF) ↑; sympathetic activity (LF) ↓; LF/HF ratio ↓.	Needle-less transcutaneous electroacupuncture at ST36 improved stress-induced gastric dysrhythmia through autonomic modulation.
Attia et al. [42]	2016	Egypt	clinical trial (intervention study)	Human	Baseline healthy control (within-subject comparison)	Traumatic brain injury (fluid percussion model)	Laser acupuncture	Three sessions per week for 4 weeks (12 sessions total)	Wavelength 904 nm; frequency 10,000 Hz; power output 100 mW; energy density 6 J/cm²	1 minute	LI4; TE5; LI11; GV14; LR3; SP6; GB34; ST36	LI4; ST36; SP6; LR3; GB34	Not reported	Not reported	TBI model: EA attenuated TNF-α expression in activated microglia and astrocytes (counts reduced; p < 0.01); IL-6, IL-1β, and CRP not reported.	Not reported	Laser acupuncture is a promising treatment modality to reduce the pain and suffering of RA patients. All RA patients showed pain VAS scores in the range (50–100 mm) and (0–40 mm), before and after laser acupuncture, respectively. Acupuncture can trigger the release of endogenous analgesic substances, e.g., endorphins.	HPA/stress axis effects	Laser acupuncture alleviates oxidative stress, including reductions in lipid peroxidation markers.	Rheumatoid arthritis disease activity: DAS28 decreased significantly after laser acupuncture (baseline 5.803 ± 0.136 to 3.643 ± 0.125, p < 0.0005).	Oxidative/antioxidative markers (MDA, SOD, GR, catalase, GSH, nitrite, nitrate), inflammatory markers (CRP, IL-6, ESR), ATP concentrations.	Disease activity (DAS28) ↓; plasma MDA ↓; serum nitrate/nitrite ↓; serum CRP ↓; plasma IL-6 ↓; GPx activity ↓; ESR ↓; plasma SOD ↑; GR ↑; catalase ↑; blood GSH ↑; plasma ATP ↑; pain VAS ↓.	Laser acupuncture reduced oxidative stress, inflammation, and disease activity in rheumatoid arthritis, supporting its therapeutic potential.
Tang et al. [41]	2016	Taiwan	mechanistic animal study	Animal	Sham operation control and TBI model control	Sepsis-induced brain injury (cecal ligation and puncture)	Electroacupuncture	Daily for 3 consecutive days	Sparse–dense wave, 0.2/1 Hz; amplitude 1 mA	60 minutes	GV20; GV26; LI4; KI1	ST36; GV20	Not reported	EA attenuated inflammation via activation of α7 nicotinic acetylcholine receptors, protecting against cerebral ischemic injury.	EA pretreatment reduced IL-6 and TNF-α in sepsis/CLP model (serum and hippocampal) across waveforms; IL-1β and CRP not reported.	TBI model: EA attenuated microglial and astrocyte activation and reduced neuronal apoptosis in peri-lesional cortex (TUNEL/Caspase-3/Neu-N markers).	TBI-induced loss of grasp strength was significantly attenuated by EA treatment. EA can increase β-endorphin content in tissues with local inflammation.	Not Reported	Not Reported	Inclined plane (motor function): TBI reduced maximal grip angle vs sham (51.1° ± 0.43° vs 57.2° ± 0.66°, p < 0.001); EA improved motor dysfunction (51.1° ± 0.43° vs 56.1° ± 1.33°, p < 0.01).	TTC infarct volume; neuronal apoptosis (TUNEL, caspase-3); microglia/astrocyte activation; TNF-α expression.	Maximal grip angle ↑; brain infarction volume ↓; neuronal apoptosis ↓; microglia activation ↓; TNF-α expression in activated microglia ↓.	Early electroacupuncture reduced neuroinflammation and promoted functional recovery after traumatic brain injury.
Chen et al. [61]	2016	China	mechanistic animal study	Animal	Sham operation control and sepsis (CLP) model control	Post-thyroidectomy incisional neck pain	Electroacupuncture (pretreatment)	Single session	2 mA with frequencies 2 Hz, 2/10 Hz, or 2/15 Hz; waveforms included continuous, intermittent, or dilatational	30 minutes	GV20; ST36	LI4; PC6; ST36; GB34	Not reported	Cholinergic anti-inflammatory reflex discussed; EA pretreatment across waveforms conjectured to exert anti-inflammatory effects via vagus nerve.	Not reported	CLP brain injury model: EA pretreatment reduced TNF-α and IL-6, reduced MDA, increased antioxidant enzymes (SOD, CAT) in serum and hippocampus at 48 h post-procedure.	EA pretreatment with different waveforms prevents brain injury in rats subjected to cecal ligation and puncture via inhibiting microglial activation. Additionally, EA pretreatment downregulated the expressions of toll-like receptor-4, nuclear factor–kappa B and ionized calcium binding adaptor molecule 1. The TLR-4/NF-κB signal pathway is involved in the pathological process of inflammation response.	Not Reported	Not Reported	EA pretreatment increased 7-day survival after CLP; DW regimen attenuated 168-h mortality (p < 0.01 vs CLP and p < 0.01 vs CW).	EA pretreatment: improved survival; reduced encephaledema/brain injury/apoptosis/cognitive dysfunction; preserved BBB; escape latency improved (DW most significant vs CLP, p < 0.01); water content and EB reduced vs CLP (p < 0.01).	Survival rate ↑; cognitive dysfunction (latency) ↓; encephaledema ↓; BBB permeability ↓; apoptosis index ↓; TLR-4/NF-κB/Iba1 protein expression ↓; MDA ↓; SOD and CAT ↑.	Electroacupuncture pretreatment protected against sepsis-induced brain injury by suppressing inflammation, oxidative stress, and apoptosis.
Qiao et al. [62]	2017	China	mechanistic animal study	Animal	Disease model control (untreated model group)	Chronic obstructive pulmonary disease	Electroacupuncture	Three sessions at 4, 24, and 48 hours post-surgery	Alternating frequency 2/100 Hz; intensity 1 mA; pulse width 0.2–0.6 ms	30 minutes	LI18; LI4; PC6; ST36; GB34	ST36	Not reported	Not reported (mechanism focused on spinal GABA/GAD67).	COPD model: EA reduced IL-6 and TNF-α in BALF and lung tissue (e.g., BALF IL-6 267.43 ± 7.28 to 188.37 ± 36.02 pg/mL; BALF TNF-α 275.54 ± 25.65 to 206.47 ± 32.02 pg/mL); IL-1β and CRP not reported.	EA analgesia mechanisms include central endorphin pathways, supraspinal serotonergic/noradrenergic descending systems, spinal GABA, and opioid neurotransmitters; study focused on cervical DRG (C3–C6) and modulation of nociceptive transmission in spinal dorsal horn.	EA modulated expression of the neuropeptides substance P and calcitonin gene-related peptide. EA at LI18 and LI4-PC6 suppressed neck incision-induced upregulation of mRNA/protein expression of SP/CGRP. EA at LI18 and LI4-PC6 upregulated mRNA/protein expression of GAD67. SP and CGRP are important molecules in nociceptive processing. SP is released by the C-type primary afferent terminals of the small DRG neurons. GAD67 expression was found in larger neurons >40 µm in diameter.	Not Reported	Not Reported	Thermal pain threshold increased after EA (LI18 and LI4–PC6 groups) vs untreated model (both p < 0.001).	SP/CGRP/GAD67 mRNA and protein in C3–6 DRGs.	Thermal pain threshold (LI18/LI4-PC6) ↑; SP mRNA/protein ↓; CGRP mRNA/protein ↓; GAD67 mRNA/protein ↑.	Electroacupuncture at LI18 and LI4-PC6 increased pain thresholds by downregulating pronociceptive mediators and enhancing inhibitory neurotransmission.
Zhang et al. [30]	2018	China	mechanistic animal study	Animal	Normal control and pharmacological blockade (α-bungarotoxin)	TNBS-induced colonic inflammation	Electroacupuncture	Once daily for 7 days	Dense–sparse alternating waves, 10/50 Hz; intensity 2 mA; pulse width 0.2–0.6 ms	30 minutes	ST36; BL13	ST36	EA enhanced vagal nerve discharge in COPD model: frequency 836.00 ± 91.33 vs 699.33 ± 16.20 Hz (p = 0.004); discharge area 155.00 ± 29.61 vs 88.50 ± 18.90 μV/s (p < 0.001); amplitude 25.44 ± 5.96 vs 13.70 ± 2.09 μV (p < 0.001).	EA regulates CAP in COPD; pathway involves vagus nerve, acetylcholine, α7nAChR, and downstream inflammatory signaling.	TNBS colitis: EA reduced plasma TNF-α, IL-1β, and IL-6 (EA2 significant); MPO activity reduced (EA2 significant vs sham-EA).	Not reported (focus on cervical vagus nerve and downstream lung tissue).	Not Reported	HPA/stress axis effects	The vagus nerve mediates anti-inflammatory effects via the hypothalamic–pituitary–adrenal axis, the cholinergic anti-inflammatory pathway, and splenic sympathetic signaling.	COPD model: EA improved lung function and vagal discharge (p < 0.01); FVC 16.32 ± 1.34 mL (COPD+EA) vs 10.90 ± 1.15 mL (COPD), p < 0.01.	COPD: lung inflammation improved; ACh, AChE, IL-6, TNF-α decreased (p < 0.01); A/S value increased after EA (p < 0.01); α7nAChR, JAK2, STAT3, NF-κB downregulated (p < 0.05 to p < 0.01).	Lung function ↑; vagal discharge ↑; lung inflammation (A/S ratio) ↑; IL-6 and TNF-α ↓; ACh and AChE ↓; α7nAChR/JAK2/STAT3/NF-κB expression ↓.	Electroacupuncture improved lung inflammation and pulmonary function in COPD via cholinergic anti-inflammatory mechanisms.
Jin et al. [63]	2019	US and China	mechanistic animal study	Animal	Sham electroacupuncture without current	Opioid-induced constipation	Electroacupuncture via chronically implanted electrodes	"The treatments lasted for 3 weeks"	EA1: 25 Hz, 4 mA; EA2: 5 Hz, 4 mA; pulse width 0.5 ms	3 hours (chronic stimulation); 30 minutes for acute experiments	ST36	ST36	EA increased vagal activity and reduced LF/HF acutely. EA1 vagal activity 0.34 ± 0.01 during EA and 0.33 ± 0.02 after vs 0.28 ± 0.02 baseline (p < 0.04). EA1 LF/HF 2.02 ± 0.24 during and 2.07 ± 0.09 after vs 2.72 ± 0.30 baseline (p < 0.04–0.05). EA2 vagal activity 0.27 ± 0.011 to 0.39 ± 0.02 during (p < 0.001) and 0.36 ± 0.01 after (p < 0.001). EA2 LF/HF 2.77 ± 0.15 to 1.61 ± 0.14 during (p < 0.005) and 1.79 ± 0.017 after (p < 0.01).	EA increased vagal activity in acute/chronic conditions; anti-inflammatory effects believed mediated by vagal efferents releasing acetylcholine in the colon and suppressing pro-inflammatory cytokines.	Not reported	Not reported (cited evidence of normalized cortical/subcortical network metrics in Crohn’s disease; no primary CNS outcomes reported in this entry).	Not Reported	HPA/stress axis effects	Endotoxin and pro-inflammatory cytokines activate afferent vagal fibers, inducing hypothalamic–pituitary–adrenal anti-inflammatory responses.	Colitis: DAI decreased with chronic EA1 and EA2 (EA2 more potent, p < 0.05); macroscopic score reduced (sham-EA 6.4 ± 0.6 to 4.9 ± 0.1 with EA1 and 4.0 ± 0.2 with EA2, p < 0.05).	Colitis: histological score reduced with EA2 (3.0 ± 0.3; p < 0.01) but not EA1 (p > 0.05); food intake increased with EA1 (days 3–6) and EA2 (days 3–8) vs sham (p < 0.001); TNF-α/IL-1β/IL-6 decreased with EA (EA2 more potent).	DAI ↓; macroscopic score ↓; histological score (EA2) ↓; vagal activity ↑; sympathetic activity ↓; pro-inflammatory cytokines ↓.	Electrical stimulation at ST36 reduced colonic inflammation through autonomic regulation of pro-inflammatory cytokines.
Wang et al. [64]	2019	USA and China	mechanistic animal study	Animal	Sham electroacupuncture without current	Postoperative recovery following knee surgery	Electroacupuncture via chronically implanted electrodes	Once daily for 7 days	25 Hz, 2 mA; pulse width 0.5 ms; train on-time 2 s, off-time 3 s	90 minutes	ST36	PC6; ST36; SP6	EA increased HF and reduced LF and LF/HF in normal and constipation models: HF 0.57 ± 0.02 vs 0.27 ± 0.02 (p < 0.05) (normal) and 0.57 ± 0.02 vs 0.32 ± 0.02 (p < 0.05) (model). LF decreased and LF/HF reduced in both conditions.	EA enhanced vagal activity; increased vagal efferent output from DMV may activate colon via pelvic nerve; vagal-cholinergic pathways referenced.	Postoperative setting: CRP significantly lower in intervention group at POD1/3/7; neutrophil/lymphocyte ratio also measured.	ST36 stimulation may activate Barrington’s nucleus via somatic-autonomic pathway; enhanced vagal efferent output activates upper gut; lop does not cross BBB (used for mechanistic inference).	Lop works by a chemical structure similar to opioid-receptor agonist and acts on the μ opioid receptors which locate at myenteric plexus of the bowel.	Gut–brain axis findings; HPA/stress axis effects	Electroacupuncture improves gastric, intestinal, and whole-gut transit through autonomic regulation and normalizes stress-induced colonic dysmotility.	Constipation model outcomes: EA normalized fecal pellet number (Lop+EA 39.2 ± 0.5 vs Lop+sham 29.8 ± 0.6, p < 0.01), distal colon transit time (9.5 ± 0.8 min vs 53.2 ± 3.4 min, p < 0.01), and whole-gut transit time (11.1 ± 0.1 h vs 13.5 ± 0.1 h, p < 0.01).	Feces characteristics; gastric emptying; small intestinal transit; HRV.	Fecal pellet number ↑; fecal pellet weight ↑; fecal water content ↑; gastric emptying ↑; small intestinal transit ↑; dCTT ↓; WGTT ↓; HF ↑; LF ↓; LF/HF ↓.	Chronic electroacupuncture at ST36 alleviated opioid-induced constipation by enhancing gastrointestinal motility via autonomic pathways.
Chi et al. [65]	2019	China	China	Human	Sham transcutaneous electrical stimulation	Cerebral ischemia–reperfusion injury	Transcutaneous electrical acupoint stimulation (TEAS)	Three sessions (pre-epidural anesthesia, plus postoperative days 1 and 2)	Alternating frequency 2/10 Hz; intensity adjusted to maximal tolerance	30 minutes	ST36; SP6; PC6; LI11	SP6; GV20	Not reported	Not reported	Inflammatory markers assessed (TNF-α, IL-6, IL-1β, CRP), but quantitative values not provided in the extracted entry.	Not reported	TEAS can trigger the release of endogenous neurotransmitters including endogenous analgesic substances, e.g., endorphins. The postoperative VAS score was shown as a lower value at first day after surgery.	HPA/stress axis effects	Perioperative stress activates hypothalamic–pituitary–adrenal and sympathetic systems; electroacupuncture reduces cortisol and ACTH levels postoperatively.	Primary endpoint QoR-40: lower in control than EA at POD1 (e.g., 160.1 ± 5.5 vs 170.9 ± 5.0, p < 0.05).	Secondary endpoints: COR, ACTH, CRP, N/L, and VAS.	QoR-40 ↑; COR ↓; ACTH ↓; CRP ↓; N/L ↓.	Perioperative transcutaneous electrical acupoint stimulation facilitated early postoperative recovery by reducing inflammation and stress responses.
Long et al. [66]	2019	China	mechanistic animal study	Animal	Sham operation control and MCAO model control	Gastric motility regulation	Electroacupuncture (pretreatment)	Single session	Continuous wave 2/100 Hz, 1 mA	60 minutes	GV20; BL23; SP6	PC6	Not reported	Not reported	Not reported	EA pretreatment reduced P38 phosphorylation; spinal cord dorsal horn phosphorylation changes are implicated in exaggerated pain after inflammation (SCDH referenced in mechanistic context).	Transient receptor potential vanilloid 1 has been indicated to take part in cerebral protection of EA. EA pretreatment suppressed TRPV-1 expression in MCAO rats. EA pretreatment curbed P38 phosphorylation. EA pretreatment inhibited the ischemia-reperfusion injury-induced mitochondrial damage and P38 MAPK activation via suppressing TRPV-1. The mechanism involves TRPV1-mediated anti-oxidant stress and anti-inflammation via inhibiting P38 MAPK activation.	Not Reported	Electroacupuncture facilitates gastric motility via the vagovagal reflex, particularly through stimulation at PC6.	MCAO model: neurological deficit scores decreased and infarct area reduced with EA vs model (infarct volumes: p < 0.001 vs MCAO).	MCAO: MDA down, GSH/SOD up vs MCAO (all p < 0.01); cytochrome C-positive hippocampal neurons reduced; P38MAPK activation prevented (p < 0.05).	Neurological deficit scores ↓; infarct volumes ↓; MDA ↓; GSH and SOD ↑; TNF-α and IL-1β ↓; TRPV-1 expression ↓; P38 phosphorylation ↓.	Electroacupuncture pretreatment mitigated cerebral ischemia-reperfusion injury through TRPV1-mediated antioxidant and anti-inflammatory effects.
Lu et al. [21]	2019	China	mechanistic animal study	Animal	Sham surgery, vagotomy, sympathectomy, and neurochemical microinjection controls	Formalin-induced acute inflammatory pain	Electroacupuncture	Single session	2/15 Hz, 2 mA	2 minutes	PC6	ST36	EA at PC6 increased parasympathetic discharge more than sympathetic; effect weakened after vagotomy.	EA at PC6 promoted gastric motility by modulating vagal efferent activity, potentially via inhibition of GABA transmission to DMV.	Formalin pain model: spinal IL-6 and IFN-γ increased and IL-4 decreased in model; EA associated with lower IL-6 and IFN-γ and higher IL-4; CRP and IL-1β not reported.	Brainstem vagovagal circuits include NTS and DMV; GABA microinjection reduced gastric motility and parasympathetic discharge, while EA at PC6 increased gastric motility and parasympathetic discharge by reversing GABA-mediated inhibition in DMV and reducing inhibitory effects on vagal efferent motor neurons.	Not Reported	Gut–brain axis findings	Not Reported	PC6 EA increased gastric motility after 30 s stimulation vs baseline (*p < 0.05, n = 8).	PC6: reversed GABA-DMV inhibition of gastric motility (*p < 0.05); promoted vagal discharge post-GABA (*p < 0.05).	Gastric motility ↑; parasympathetic nerve discharge ↑; sympathetic nerve discharge: no significant increase; GABA injection reduced gastric motility (↓); EA + GABA reversed inhibition (↑).	Electroacupuncture at PC6 restored gastric motility by relieving GABA-mediated inhibition of efferent vagal activity.
Liu et al. [26]	2019	China	mechanistic animal study	Animal	Disease model control (no electroacupuncture)	Chronic stable angina pectoris	Electroacupuncture (preemptive analgesia)	Single session	2 Hz, 1 mA	30 minutes	ST36; BL40	LI4; PC6; ST36; SP6; LR3	Not reported	Not reported (analgesic effects linked to endogenous opioids/cytokines/central neurotransmitters).	CSAP: acupuncture mechanisms described as reducing CRP and TNF-α (no quantitative values provided in extracted entry).	Formalin model: Iba1, c-fos, proinflammatory cytokines, and pain neurotransmitters increased in L4–L5 spinal cord; pre-EA attenuated nociceptive effects and reduced spinal microglia and neuron activation; EA acts via peripheral, spinal, and supraspinal mechanisms.	Pain neurotransmitters, substance P and calcitonin gene-related peptide, were dramatically increased. Pre-electroacupuncture significantly attenuated spinal microglia and neurons activation, proinflammatory cytokines and pain neurotransmitters upregulation. EA dramatically decreased the activation of microglia and neurons by decreasing the expression of IFN-c, IL-6, SP, and CGRP, thus interrupted the crosstalk between microglia and sensory neurons in spinal cord. SP: Model ≈ 72 pg/ml, Pre-EA ≈ 42 pg/ml. CGRP: Model ≈ 42 pg/ml, Pre-EA ≈ 26 pg/ml.	Not Reported	Not Reported	Formalin pain: pre- and post-EA reduced paw flinching and lifting time (p < 0.05); phase 2 cumulative flinching model ≈ 150 vs post-EA ≈ 92 vs pre-EA ≈ 80; phase 2 cumulative licking model ≈ 110 s vs post-EA ≈ 75 s vs pre-EA ≈ 65 s.	Formalin model: pre-EA more effective than post-EA; reduced flinching/licking; decreased Iba1 and c-fos (p < 0.05); pre-EA reduced IL-6/IFN-γ and increased IL-4 vs post-EA (p < 0.05).	Paw flinching ↓; licking time ↓; Iba1 ↓; c-fos ↓; IL-6 and IFN-γ ↓; IL-4 ↑; SP and CGRP ↓.	Pre-electroacupuncture provided effective preemptive analgesia by suppressing inflammatory and nociceptive signaling.
Yu et al. [67]	2020	China	RCT	Human	Sham acupuncture or no-treatment control	Essential hypertension	Acupuncture or electroacupuncture	Not reported (This is a data mining study summarizing prescriptions, not a primary trial).	Not reported	Not reported	PC6; LU9; ST36; BL15; HT7; LR3; SP6; LI4; ST40; RN17	LI4; ST36; SP6; LR3	Review-level statement: acupuncture may increase HRV in CSAP and improve cardiac autonomic function (no primary numeric HRV results reported here).	Not reported	Not reported	Cardiac sensory afferents located C8–T5; overlap of meridian-related sensory and sympathetic ganglion regions proposed as mechanism linking heart/pericardium meridians to cardiac autonomic regulation.	Not reported.	Not Reported	Not Reported	Not reported (systematic review; primary outcomes varied across included trials such as angina attack frequency, VAS, SAQ, rescue meds, exercise tests, HRV, ST-segment depression, QoL).	Not reported	HRV ↑; ET ↓; NO ↑; MMP-9 ↓; NLR ↓; CRP ↓; TNF-α ↓.	Network analysis identified PC6, LU9, and ST36 as core acupoints for stable angina, potentially acting through autonomic and inflammatory regulation.
Huang et al. [68]	2020	Taiwan	RCT	Human		Neurodegenerative and neurological disorders	Manual acupuncture (De Qi–elicited)	Twice weekly for 12 consecutive weeks	Not reported	30 minutes	SP10; SP6; LR3; ST36; LI4	LI4; PC6; ST36; GV20	12-week trial: between-group SDNN (p = 0.008) and TP (p = 0.04). Within-group: SBP decreased 8.06 mmHg; DBP decreased 4.81 mmHg. LF and HF changes reported (ranges provided), with HF as parasympathetic and LF as sympathetic proxies.	Not reported	Review/perspective: acupuncture discussed as regulating IL-1β, TNF-α, and IL-6; some studies report reduced peak TNF-α with GV20/ST36.	LR3 described as improving hypertension by altering brain activation in spontaneously hypertensive rats.	Not Reported	Not Reported	Not Reported	Between-group differences observed in SBP (p = 0.001), SDNN (p = 0.008), and total power (TP) (p = 0.04).	Secondary outcome was CCMQ score; within-group improvements noted for qi deficiency (p = 0.003), blood stasis (p = 0.02), and qi depression (p = 0.03).	SBP ↓; DBP ↓; SDNN ↑; LF ↑; HF ↑; TP ↑.	Combined antihypertensive therapy and acupuncture improved blood pressure control and autonomic balance more effectively than medication alone.
Wei et al. [69]	2020	Taiwan	SLR	Human and animal	Functional dyspepsia	Manual acupuncture and electroacupuncture (including TAES)	Not reported (varied across included studies)	Not reported	Varied across studies	GV20; GV14; ST36; ST37; LI4	ST36	Stimulation induced initial pressor response (BP↑, HR↓) with longer-term BP reduction; LF/HF referenced (no extractable primary numeric summary provided here).	CAP described as α7 AChR-dependent; EA hypothesized to activate CAP via vagus nerve (acetylcholine transmitter).	Not reported	Acupuncture improves consciousness/cognition in nervous system diseases; CNS structures implicated include frontal lobe, hippocampus, ischemic regions, SCDH, and limbic system; pathways include p38 MAPK and Raf/MAPK/ERK1/2 signaling.	Mechanisms involve regulation of ion channels, scaffold proteins, and transcription factors including TRPV1/4, Nav, BDNF, and NADMR1. EA exerts analgesic effects through the interference of both the ascending and descending pain signaling pathway. EA relieves acute pain through the release of opiates to activate µ-, δ-, and κ-opioid receptors. EA was found to modulate expression of TRPV1 and TRPV4. EA relieves pain by downregulating microglial activation and reducing markers like astrocytic marker glial fibrillary acidic protein and microglial marker ionized calcium-binding adapter molecule 1.	HPA/stress axis effects	Electroacupuncture at GV20 and ST36 reduces peak adrenocorticotropic hormone and heat shock protein-70 levels under stress.	Not reported (systematic review; no single primary outcome for the review itself).	General secondary outcomes (review-level): improved consciousness/cognition; symptom/function improvement in nervous system diseases; analgesia; anti-apoptosis; reduced postoperative analgesic requirements.	Dual regulation noted: p38 MAPK pathway activity ↓ (AD/PD context); cerebral blood flow velocity (CBFV) ↑ (stroke context).	Acupuncture exerts dual regulatory effects through differential modulation of p38 MAPK pathways across neurological conditions.	
Zhang et al. [44]	2020	USA	mechanistic animal study	Animal	Sham electroacupuncture without current	COVID-19–associated systemic inflammation	Electroacupuncture	Acute, single-session experiments conducted on separate days	25 Hz, 4 mA; train on-time 2 s, off-time 3 s	30 minutes	ST36	PC6; ST36; GV20	In FD model under stress, EA increased HF (p = 0.002) and reduced LF/HF (p = 0.004) versus sham; stress reduced HF and increased LF/HF in sham sessions (values reported).	EA effects mediated via vagal efferent pathway; acetylcholine release in stomach; atropine suppressed EA effects on gastric slow waves, supporting cholinergic mediation.	CAP described as inhibiting TNF, IL-1β, and IL-6 (no quantitative values).	FD rats: EA increased c-Fos–positive cells in NTS (p < 0.05); NTS is a key target of vagal afferents; mechanism described as activated peripheral nerves acting on NTS neurons.	Not Reported	Gut–brain axis findings; HPA/stress axis effects	Electroacupuncture at ST36 improves stress-induced gastric dysrhythmia, increases acetylcholine levels in the stomach and duodenum, and suppresses stress-related norepinephrine elevation.	EA improved gastric slow waves during stress; percentage of normal GSW higher with EA than sham (p < 0.05).	Mechanistic secondary outcomes: increased HF (vagal activity), reduced LF/HF, suppressed plasma NE, increased c-Fos in NTS, increased ACh in stomach/duodenum.	Percentage of normal GSW ↑; HF ↑; LF/HF ↓; plasma NE ↓; NTS c-Fos ↑; ACh in gastric antrum/duodenum ↑.	Electroacupuncture improved gastric slow waves under stress via central autonomic circuits involving the NTS and vagal efferents.
Qin et al. [70]	2020	China	PERSPECTIVE report	Human/Animal (Proposed Strategy).	Not applicable (conceptual comparison with standard drug therapies)	Post–cesarean section gastrointestinal dysfunction	Acupuncture combined with vagus nerve stimulation	Not Reported	Not reported (conceptual/proposed parameters only)	Not reported	ST36; PC6; GV20; BL13	ST36	Not reported	Inflammatory reflex/CAP described: efferent vagus → splenic nerve → norepinephrine → ChAT+ T cells → acetylcholine → α7nAChR on macrophages → cytokine suppression; acupuncture proposed to activate CAP via vagal pathways.	Postoperative TEA: no between-group differences in TNF-α or IL-6 on POD1/POD3; IL-6 decreased from POD1 to POD3 in both groups.	Auricular VNS: afferent vagal signals engage CNS and activate opposing efferent vagus output; auricular concha has connections relevant to efferent vagal regulation.	Not reported	Gut–brain axis findings	Not Reported	Not reported (perspective article proposing a strategy).	Conceptual outcomes: mitigating “cytokine storm”; improved survival in inflammatory diseases; COVID-19 control (conceptual).	Not reported	Activation of the cholinergic anti-inflammatory pathway represents a feasible therapeutic strategy against cytokine storm, including COVID-19.
Li et al. [71]	2020	Chin and USA	RCT	Human	Sham transcutaneous electrical acustimulation at non-acupoint	DSS-induced ulcerative colitis	Transcutaneous electrical acustimulation (TEA)	TEA: one hour on POD0, then twice daily POD1–POD3 (7 sessions total)	25 Hz; pulse width 0.5 ms; train on-time 2 s, off-time 3 s; intensity 2–10 mA	60 minutes	ST36	ST36	TEA increased vagal activity and decreased sympathetic activity on POD4 vs POD0 (both p = 0.013). HF increased (0.44 ± 0.15 vs 0.38 ± 0.14; p = 0.013). LF/HF decreased (0.56 ± 0.15 vs 0.62 ± 0.14; p = 0.013).	Postoperative sympathetic predominance inhibits GI motility; EA at ST36 proposed to activate NTS/DMV and enhance vagal efferent flow to GI tract.	DSS colitis: EA improved CRP and IFN-γ and reduced TNF-α (3.04 to 2.29 pg/mL) and IL-6 (26.08 to 10.83 pg/mL); IL-1β not reported.	EA at ST36 activates NTS/DMV via somatic afferents and enhances vagal efferent flow to GI tract; pain modulation described through peripheral, spinal, and supraspinal pathways.	TEA reduced VAS pain score from POD1 to POD3, p < 0.001. EA inhibits pain through peripheral, spinal, and supraspinal pathways with the involvement of a series of bioactive molecules including opioids, serotonin, norepinephrine, cytokines, signal molecules and so on.	Gut–brain axis findings	Transcutaneous electroacupuncture increases normal gastric slow waves postoperatively and promotes gastrointestinal motility via autonomic mechanisms.	Primary outcome: time to first defecation after surgery; TEA reduced time to first defecation (p < 0.001).	Post-CS recovery endpoints: time to first flatus, resume semifluid diet, ambulation, BSFS of first stool, SBMs within 4 days, glycerin enema use, VAS pain, and GI symptom scores POD1–POD3.	Time of first defecation ↓; time of first flatus ↓; BSFS of first stool ↑; number of SBMs ↑; VAS pain ↓; HF ↑; LF/HF ↓.	Needleless transcutaneous electroacupuncture at ST36 improved postoperative gastrointestinal recovery by enhancing vagal tone and suppressing sympathetic activity.
Liu et al. [72]	2020	Taiwan	mechanistic animal study	Animal	Disease model control (DSS-induced colitis)	Concanavalin A–induced hepatitis	Electroacupuncture	Nine sessions (once daily, days 5–13)	2 Hz, 1 mA; visible local muscle contraction	15 minutes	ST36	ST36	Not reported	IBD: cholinergic anti-inflammatory reflex cited as supportive (limited but present) evidence for acupuncture/EA mechanisms.	ConA hepatitis: TNF-α induced in hepatic cells and plasma; MA/EA at ST36 reduced plasma TNF-α (e.g., ~830 to ~100 pg/mL at 15 mg/kg ConA); IL-6, IL-1β, and CRP not reported.	Hypothalamic-pituitary-adrenal axis proposed contributor to anti-inflammatory effect.	Not Reported	Gut–brain axis findings; HPA/stress axis effects	Electroacupuncture modulates gut inflammation via TLR4–MyD88 signaling, improves mucosal barrier integrity, and alters gut microbiota composition.	DSS colitis: colon length higher with EA (7.71 ± 0.65 cm) vs DSS (5.53 ± 0.56 cm), p < 0.05; DAI lower with EA (0.73 ± 0.13) vs DSS (1.78 ± 0.35), p < 0.05.	DSS colitis: histopathology score improved (12.83 ± 3.54 DSS vs 5.33 ± 2.34 DSS+EA); adiponectin increased with EA; CRP and IFN-γ improved vs DSS (p ≈ 0.004).	DAI ↓; colon length ↑; CRP/IFN-γ/TNF-α/IL-6 ↓; adiponectin ↑; claudin-1 and ZO-1 preserved (↑).	Electroacupuncture exerted therapeutic effects in colitis by modulating TLR4-dependent inflammation, gut barrier integrity, and microbiota balance.
Lim et al. [31]	2020	Republic of Korea	mechanistic animal study	Animal	Disease model control and vagotomy	Mixed clinical case scenarios (musculoskeletal, psychiatric, internal medicine)	Manual acupuncture or electroacupuncture	Single session	EA: 1 Hz, 1.0 V, pulse duration 2 ms; MA: manual rotation every 5 min	30 minutes	ST36	LI4; PC6; ST36; SP6; LR3; GV20; GB34	Not reported	Hepatic inflammation: acupuncture anti-inflammatory effects modulated through vagus nerve activation; vagotomy/VNX markedly suppressed effects in ConA model.	Not reported	ConA model: AMPA receptor duration increased in dorsal motor vagal neurons via NTS stimulation; sciatic afferents transmit ST36 signals to spinal cord and brain.	Not Reported	Not Reported	Not Reported	ConA hepatitis model: MA and EA at ST36 decreased plasma TNF-α vs ConA (p < 0.001).	ConA model: phospho-ERK1/2 downregulated by MA/EA; vagotomy largely negated anti-inflammatory effects.	TNF-α production ↓; phospho-Erk1/2 ↓; CD11b/CD68 ↓.	Hepatic anti-inflammatory effects of acupuncture were mediated through vagal nerve activation.
Leee al. [73]	2020	Republic of Korea	network analysis and hierarchical clustering study	Human	Not reported	Mixed pain syndromes (visceral and somatic pain)	Not applicable (virtual acupuncture prescription analysis)	Not Reported	Not reported	Not reported	ST36; LI4; LR3	LI4; PC6; ST36; SP6; LR3; GV20; GB34; CV6	Not reported	Not reported	Not reported (referenced prior evidence of anti-inflammatory/immune regulation in OA/disc herniation contexts).	Not reported	Not Reported	Not Reported	Not Reported	Prescription-probability analysis: highest point probabilities included ST36 (0.100), LI4 (0.091), LR3 (0.078), SP6 (~0.061), PC6 (~0.057), CV12 (~0.056).	Network analysis: eigenvector centrality highest for ST36 (1.00), LI4 (0.99), LR3 (0.98), SP6 (0.82), PC6 (0.79); clustering yielded three symptom/disease clusters.	Not applicable (descriptive prescription pattern study).	Clinical data mining revealed shared and condition-specific acupoint selection patterns across acupuncture practice.
Hwang et al. [17]]	2021	Republic of Korea and USA	SLR	Human	Not applicable (point-frequency analysis study)	Mild hypertension (prehypertension or stage I hypertension)	Manual acupuncture or electroacupuncture	Highly variable across included RCTs	Highly variable across included RCTs	Not reported	SP6; ST36; LI4; LR3	PC6; ST36; LR3; GV20	Not reported	Not reported	Not reported	Neuromodulation framework: acupuncture acts via peripheral stimulation with local, segmental, and extra-segmental neuromodulation; distal points activate endogenous descending pain modulation centers; segmental inhibition via inhibitory interneurons; ST36 can activate limbic system and default mode network.	Needles are inserted locally to segmentally link pain areas that may exert local antidromic axon reflexes or inhibit the nociceptive pathway at the dorsal horn of the spinal cord. The viscerosomatic reflex can inhibit visceral pain by converging two afferents from the viscera and somatic structures into a single dorsal horn pathway. The analgesic effect of acupuncture is also noted to be associated with neuropeptides SP and CGRP.	Not Reported	Not Reported	Not applicable (systematic review of point-use frequency, not clinical efficacy outcomes); common points across diseases included SP6, ST36, LI4, LR3 (e.g., SP6 77% for dysmenorrhea).	Point-frequency summaries by condition (e.g., migraine: GB20 82%, LR3 64%, GV20 55%, Taiyang 55%, LI4 46%, TE5 46%; dysmenorrhea: SP6 77%, CV4 47%, SP8 47%, LR3 33%, BL32 33%).	Not applicable (descriptive point-selection pattern study).	Pain control in acupuncture research predominantly involves combined local and distal neuromodulation strategies.
Kimura et al. [74]	2021	Japan	Clinical Trial	Human	Baseline clinical comparator group	Dry eye disease	Manual acupuncture (De Qi–elicited; parameters not specified)	Single session	Not reported.	15 minutes	PC6; LI4; ST36; LR3; GV20	LI4; ST36; SP6; LR3; GV20	Hypertensive group: baseline HF 161.1 ± 6.9 ms²; baseline LF/HF 5.0 ± 1.2. LF/HF decreased during acupuncture (60.3% ± 6.1%; p < 0.05). HF increased during recovery (221.4% ± 38.9% vs baseline; p < 0.05).	Not reported	General anti-inflammatory effects reported (decreased pro-inflammatory cytokines), without specific markers quantified in the extracted entry.	Not reported	Not Reported	Not Reported	Not Reported	Hypertension: SBP and HR decreased during and after acupuncture vs baseline (p < 0.05); LF/HF reduced during acupuncture (p < 0.05); HF increased after acupuncture (p < 0.05).	Not reported	SBP ↓; HR ↓; LF/HF ↓; HF ↑.	Acupuncture reduced blood pressure and heart rate in mild hypertension by suppressing sympathetic tone and enhancing vagal activity.
Lin et al. [75]	2021	Taiwan	Association Rule Analysis	Human	Standard medical therapy comparator	Mixed pain syndromes	Manual acupuncture	Not reported (Data mining study).	Not reported	Not reported	TE23; BL2; ST2; ST1; EX-HN5; BL1; LI4; ST36; SP6; KI3	LI4; PC6; ST36; SP6; LR3; GV20; GB34; CV6	Not reported	Not reported	Not reported	Not reported	DED symptoms include ocular pain and acupuncture improves scratchiness.	HPA/stress axis effects	Sex steroid levels, particularly estrogen, may contribute to dry eye disease mechanisms.	Not reported (data mining; original RCT outcomes included TBUT and Schirmer I test).	Quality-of-life objective statement: reducing symptoms and objective signs to improve QoL.	Tear breakup time (BUT) ↑; Schirmer’s I test ↑; proinflammatory cytokines ↓.	Combined TE23, LI4, and ST1 represented a core acupoint set for dry eye disease treatment.
Liu et al. [12]	2021	USA and China	mechanistic animal study	Animal	Sham electrical stimulation and vagotomy	Healthy volunteers	Electroacupuncture and opto-acupuncture	Single session	10 Hz; 0–3 mA; pulse width 50 μs	15 minutes	ST36; ST25; LI10; BL56	PC6	Optical stimulation at ST36 increased left cervical vagal nerve discharge ~15-fold (per electrophysiology).	Low-intensity stimulation at ST36 (but not ST25) drives the vagal–adrenal anti-inflammatory axis; PROKR2ADV neurons required for this ST36-linked pathway.	Not reported (photobiomodulation discussed as reducing inflammation; no specific markers provided).	Low-intensity ST36 stimulation required PROKR2ADV sensory neurons to activate hindbrain vagal efferent neurons in DMV; spinal ascending projections to NTS with outputs to DMV; optical ST36 stimulation increased Fos in lamina I spinal neurons labeled from NTS; PROKR2ADV neurons project mainly to superficial dorsal horn laminae.	Not Reported	HPA/stress axis effects	Low-intensity electrostimulation at ST36 induces vagus-dependent catecholamine release from adrenal chromaffin cells.	LPS survival: 0.5 mA ES at ST36 increased survival vs 0 mA (**p = 0.008); reduced TNF (**p = 0.003) and IL-6 (**p = 0.002).	PROKR2ADV-Abl: DMV Fos induction eliminated; 0.5 mA ST36 increased NA/A/DA in controls but not ablated mice (p < 0.001).	Survival rates ↑; TNF/IL-6 production ↓; catecholamine release (NA, A, DA) ↑; Fos induction in DMV ↑.	Selective acupoint stimulation activated a vagal–adrenal axis, providing a neuroanatomical basis for acupoint specificity.
Chang et al. [76]	2022	Taiwan	Pilot Study	Human	Inactive energy output control (0 J/cm²)	Chronic pain with comorbid depression	Laser acupuncture	Each subject received all three different EDs in a randomized order	Pulsed wave 1199 Hz (Bahr 2); output power 100 mW	20–60 seconds (depending on experimental set)	PC6	GV20	Energy density study: HF% increased and LF/HF decreased at ED 7.96 J/cm²; LF% decreased vs ED 0 and ED 23.87 (p = 0.041 and p = 0.011). LF/HF differed overall (F = 3.578; p = 0.034) and vs ED 0 (p = 0.040).	Laser acupuncture may shift ANS toward increased parasympathetic and decreased sympathetic activity.	Mechanistic discussion includes pro-inflammatory cytokines (no specific markers/values provided in extracted entry).	HRV indexes reflect heart–brain interactions and ANS dynamics (neuro-cardiac function).	Not reported.	Gut–brain axis findings	PC6 stimulation activates parasympathetic pathways involved in gastric regulation.	Not reported (observational HRV-watch study; key differences in HF%, LF%, LF/HF across energy densities, e.g., LF% p = 0.011 and LF/HF p = 0.040 for specified comparisons).	Hemodynamics/HRV suite: SBP, DBP, HR, HRV, RMSSD, PNN50, TP, VLF, LF, HF.	HF% ↑; LF% ↓; LF/HF ratio ↓ (7.96 J/cm²); at 23.87 J/cm² the opposite was observed (HF% ↓; LF/HF ↑).	Laser acupuncture at PC6 modulated HRV in an energy-dependent manner, with low doses favoring parasympathetic activation.
Li et al. [77]	2022	China	RCT-single blind	Human	Active drug comparator (citalopram)	Bile duct ligation–induced cholestatic liver injury	Transcutaneous auricular vagus nerve stimulation combined with cranial electroacupuncture	taVNS twice daily for 8 weeks; EA three sessions/week for 8 weeks	taVNS: dilatational wave 4/20 Hz; EA: continuous wave 2 Hz; intensity adjusted to comfort level	30 minutes	GV20; GV29	ST36	Not reported	taVNS may signal via vagal afferents to the nucleus of the solitary tract to regulate body function.	BDL model: EA at ST36 reduced plasma IL-1β, IL-6, LPS, and TNF-α and increased IL-10 (quantitative values referenced in figure); CRP not reported.	taVNS transmits to NTS via vagal afferents; GV20/GV29 stimulation engages trigeminal sensory pathways; therapeutic mechanisms described at peripheral, spinal, and central levels.	Neurotransmitters such as glutamate, substance P, serotonin, norepinephrine, dopamine, BDNF, and gamma-aminobutyric acid are activated in CP.	Not Reported	Electroacupuncture at ST36 improves intestinal barrier integrity, increases tight junction protein expression, and suppresses NF-κB–mediated inflammation.	Primary outcome MADRS: adjusted between-group difference at week 8 was −0.47 (95% CI −2.33 to 1.39), p = 0.619.	Patient-reported measures: SF-MPQ, SF-36, PSQI, HAMD, HAMA.	MADRS ↓; SF-MPQ ↓; SF-36 BP score showed larger reduction with citalopram at 6 weeks (↓); PSQI sleeping medication use ↓ in taVNS group (vs citalopram at week 6).	
Lei et al. [78]	2022	China	mechanistic animal study	Animal	Sham electroacupuncture at ST36 and pharmacological blockade (α7nAChR)	Diarrhea-predominant irritable bowel syndrome	Electroacupuncture	Not Reported	50 Hz, 2 mA; stimulation induced visible limb tremor	45 minutes	ST36	LI4; ST36	Not reported	EA at ST36 inhibited BDL-related intestinal mucosal damage and liver fibrosis by activating HO-1–linked cholinergic anti-inflammatory signaling in intestinal tissues.	TEA study: IL-6 and IL-10 not significantly altered; TNF-α, IL-1β, and CRP not reported.	EA at ST36 can activate dorsal vagal complex and CAP via vagal system, reducing GI proinflammatory cytokine production.	Not Reported	Gut–brain axis findings	Acupuncture modulates the brain–gut axis and visceral hypersensitivity, including serotonergic signaling in colonic mucosa.	BDL model: EA at ST36 reduced plasma IL-1β, IL-6, LPS, TNF-α and increased IL-10 (p < 0.05 to p < 0.001); intestinal injury (Chiu) scores improved (p < 0.001).	BDL: AST/ALT reduced; barrier permeability improved; fibrosis reduced; α7nAChR and HO-1 upregulated; NF-κB activation attenuated.	IL-1β/IL-6/TNF-α ↓; IL-10 ↑; ALT/AST ↓; tight junction proteins (ZO-1, occludin, claudin-1) ↑; NF-κB activation ↓.	Electroacupuncture at ST36 reduced inflammation, intestinal barrier damage, and liver fibrosis through α7nAChR-mediated NF-κB inhibition.
Hu et al. [32]	2022	China and USA	RCT	Human	Sham transcutaneous electrical acustimulation	Chronic pelvic pain syndrome	Transcutaneous electrical acustimulation (TEA)	one month (twice daily)	ST36: 25 Hz; LI4: 100 Hz; pulse width 0.5 ms; intensity tolerable to patient	60 minutes	LI4; ST36	SP6	Autonomic biomarkers (NE, PP) not significantly altered with TEA; change in NE correlated inversely with improvement in abdominal pain (correlation direction noted; no HRV change).	TEA autonomic–cytokine pathway proposed: peripheral afferents → CNS processing → increased vagal efferent output → acetylcholine release in gut → α7nAChR-mediated cytokine balancing (hypothesized, not proven in-study).	CPPS model: prostatic TNF-α, IL-1β, and PGE2 measured and reduced with EA; IL-6 and CRP not reported (values provided in entry).	IBS-D mechanisms include brain–gut effects; acupuncture associated with reduced abdominal pain and altered central functional connectivity/segregation in cited work.	Not Reported	Gut–brain axis findings	Not Reported	Abdominal pain: both TEA and sham reduced pain, but TEA had greater effect (p = 0.014; VAS change 3.5 ± 2.00 vs 1.0 ± 1.88).	IBS trial: TEA improved QoL (78.55 ± 9.62 to 85.97 ± 9.49, p < 0.0001); both TEA and sham improved IBS-SSS (TEA p < 0.0001; sham p = 0.004); dropout higher in sham (29% vs 0%, p = 0.021).	Abdominal pain (VAS) ↓; IBS-QOL ↑; IBS-SSS ↓; drop-out rate ↓.	Transcutaneous electroacupuncture improved abdominal pain and quality of life in IBS-D via non-autonomic mechanisms.
Chang et al. [39]	2022	China	mechanistic animal study	Animal	Disease model control	Lyme disease–associated inflammatory arthritis	Electroacupuncture	once daily for four therapeutic courses (5 d as a course), 2 d break between the two courses	Dense wave 2/100 Hz; intensity not reported	Not reported	CV4; CV3; BL35; SP6	ST36	Mechanistic statement: parasympathetic innervation overlap and parasympathetic regulation proposed for pelvic/prostate-related points (no HRV metrics).	Not reported	Lyme arthritis model: EA reduced IL-17A/IL-17F/IL-22 and also reduced TNF-α and IL-2; IL-1β, IL-6, and CRP not reported.	SP6 stimulation can transmit via tibial nerve to dorsal roots/spinal cord and regulate organ function via central feedback.	The expression levels of purinergic 2X7 receptor, NOD-like receptor pyrin domain-containing 3, caspase-1 and interleukin-18 mRNA were quantified. EA at Guanyuan, Zhongji, Huiyang and Sanyinjiao can produce anti-inflammatory analgesia effect by preventing the activation of P2X7R/NLRP3 signal pathway, inhibit the release of inflammatory cytokines in CPPS rats. When NLRP3 inflammasome is activated, ASC and caspase-1 can be recruited, and then the inactive precursor caspase-1 can be transformed into active caspase-1 by cutting its precursor, so as to activate the downstream pathways and promote the secretion and release of IL-1β, IL-18 and PGE2. The activated P2X7R can also activate the Egr transcription factor through the ERK/P38 MAPK signaling pathway, which induces the production of TNF-α.	Not Reported	Not Reported	Chronic pain model: after 3rd course EA rescued pain threshold vs model (p < 0.01); after 4th course EA 10.0 ± 0.7 s vs model 5.4 ± 0.6 s.	CPPS: prostate index decreased (EA 1.19 ± 0.09); P2X7R/NLRP3 downregulated; histology improved; PI and prostate wet weight reduced (p < 0.01).	Thermal pain threshold ↑; prostatic index ↓; TNF-α/IL-1β/PGE2 ↓; P2X7R/NLRP3/caspase-1/IL-18 mRNA ↓.	Electroacupuncture alleviated inflammatory pain in CPPS by suppressing the P2X7R/NLRP3 signaling pathway.
Akoolo et al. [23]	2022	USA	mechanistic animal study	Animal	Mock-treated control (non-conductive needles)	Drug-resistant epilepsy	Electroacupuncture	Treatments on days 0, 1, 2, 3, 4, 5, 7, 8, 9, 11, and 13 (11 sessions/14 days)	10 Hz; 4 V AC; 40 mA; pulse width 50 μs	10 minutes	ST36	GV20; GB34	Mechanistic statement: sciatic–vagal network signaling induces catecholamine changes; dopamine production reported (no HRV metrics).	EA at ST36 activates the sciatic–vagal network to control systemic inflammation; dopamine production implicated in anti-inflammatory activity; vagus nerve as key inflammation regulator.	Thread-embedding acupuncture: prolonged exposure described as promoting an anti-inflammatory response (no specific markers quantified).	Sciatic nerve EA neuromodulates brain and signals adrenal activation through vagus to produce dopamine (sciatic–vagal/adrenal pathway).	Acupuncture has been used for a long time in Asian medicine to diminish pain during various ailments. Dopamine D2 receptor is involved in alleviation of Type II Collagen-Induced Arthritis in Mice.	Not Reported	Not Reported	Lyme arthritis: mediolateral joint swelling reduced with EA (mock median ≈ 4.1 mm vs EA median ≈ 3.5 mm, p < 0.0001).	Lyme arthritis: leukocyte infiltration reduced with EA; neutrophil infiltration reduced; splenic IL-22 reduced; Th1/Th17 infiltration reduced (*p < 0.05, **p < 0.01).	Joint swelling ↓; leukocyte infiltration ↓; proinflammatory cytokines ↓; Th1 and Th17 infiltration ↓.	Electroacupuncture reduced inflammatory activity and improved outcomes in chronic Lyme disease models.
Nguyen et al. [79]	2023	Vietnam	RCT	Human	Sham transcutaneous electrical acustimulation	Knee osteoarthritis	Thread-embedding acupuncture	Four sessions of TEA or sham TEA spaced 4 weeks apart	Not reported	Not reported	GV20; GV14; BL15; BL18; ST40; GB34	ST36; GB34	RMSSD reported as vagus-mediated HRV measure: RMSSD (median, IQR) 33.73 (24.6–46.14). LF range 0.04–0.15 Hz; HF range 0.15–0.40 Hz.	VNS is a standard DRE treatment (vagus stimulation). TEA mechanism proposed: increased RMSSD suggests increased parasympathetic (vagal) activity.	KOA (MIA) model: EA reduced synovial TNF-α, IL-1β, and IL-6 on day 9 (approximate values provided from figure).	DRE mechanisms: thalamus implicated in seizure propagation; GV14 inhibits epileptiform activity in ventrobasal thalamic neurons; GB34 modulates thalamic gene expression; additional mechanisms include reduced hippocampal apoptosis via P53 inhibition and increased bcl-2.	TEA mechanism encompasses alterations in nerve potential and membrane ion channels.	Not Reported	Not Reported	Seizure control: satisfactory control at T4 higher with TEA than sham (37% vs 3.7%, p = 0.003).	Seizure outcomes set: satisfactory control, seizure freedom, HR and HRV; RMSSD increased in TEA (p = 0.008).	HR ↓; HRV ↑; RMSSD ↑.	Transcutaneous electroacupuncture improved seizure control and autonomic balance in drug-resistant epilepsy.
Chen et al. [80]	2023	China	mechanistic animal study	Animal	Disease model control (MIA-induced osteoarthritis)	Ligature-induced periodontitis	Electroacupuncture	Every other day during the midterm inflammatory pain phase (day 5–9)	Dense–sparse wave 2/15 Hz, 1 mA	30 minutes	ST36; GB34	ST36	Not reported	Not reported (focus on sympathetic signaling; vagal immune modulation noted as emerging).	Periodontitis model: EA reduced IL-1β, IL-6, IL-17, and TNF-α; increased IL-4, IL-10, and IL-22 in periodontal tissues.	KOA model: EA increased c-fos + TH neurons in LC and RVLM (LC p < 0.01; RVLM p < 0.001); PVN did not participate; CXCL1/CXCR2 axis linked to MAPK signaling (MEK/ERK).	EA alleviated inflammatory pain behaviors and relieved referred hind paw pain. EA inhibited synovial macrophages. EA prevented saphenous nerve demyelination. Pain relief pathway: EA suppressed CXCL1-CXCR2-dependent overexpression of IL-6 in the synovial macrophages to relieve referred hind paw pain by activating the sympathetic noradrenergic signaling.	HPA/stress axis effects	Plasma corticosterone levels and PVN c-Fos activation were unchanged, indicating no measurable hypothalamic–pituitary–adrenal activation in this model.	KOA pain behaviors: midterm EA improved weight-bearing deficit (days 7 and 9) and increased 50% HPWT (day 14) vs model (p values as reported in figure annotations).	KOA: cartilage damage score; synovial cytokines; synovial hyperplasia/inflammatory infiltration.	WBD ↓; HPWT ↑; cartilage damage score ↓; synovial NE ↑; synovial CXCL1/IL-6/TNF-α/IL-1β ↓; circulating monocytes ↓.	Midterm electroacupuncture exerted localized anti-inflammatory effects in osteoarthritis without increasing systemic catecholamines.
Liu et al. [81]	2023	Chongqing, China; Beijing, China. The study site locations include the "College of Stomatology, Chongqing Medical University" and "Neuroscience Research Institute, Peking University".	mechanistic animal study	Animal	Disease model control (ligature-induced periodontitis)	Inflammatory bowel disease (DSS-induced colitis)	Electroacupuncture	Every other day from day 7 to day 19 (7 sessions inferred from timeline)	10 Hz, 1 mA	20 minutes	ST36	ST36	Not reported	EA regulates distant physiology via somatic sensory–autonomic reflexes and systemic anti-inflammatory neuroendocrine–immune pathways.	EA reduced serum IL-1β, IL-6, TNF-α, PGE2 and increased IL-10 (anti-inflammatory) in the extracted study entry.	Not reported (references neuro–endocrine–immune pathway but no specific CNS effects extracted here).	EA suppressed the infiltration of macrophages and neutrophils. EA also regulated genes associated with macrophage polarization: iNOS and Arg-1. The ratio of Arg-1 to iNOS was decreased in the LIP group and up-regulated in the LIP+EA group.	Gut–brain axis findings; HPA/stress axis effects	Electroacupuncture modulates the oral microbiome and exerts systemic anti-inflammatory effects via neuroendocrine–immune pathways.	Periodontitis: EA reduced alveolar bone resorption (e.g., mesial CEJ–ABC 0.282 ± 0.044 mm vs 0.347 ± 0.045 mm, p < 0.01).	Periodontitis: reduced macrophages/neutrophils; periodontal microbiome regulation; cytokine gene expression changes (IL-1β, IL-6, IL-17, TNF-α, IL-4, IL-10, IL-22).	Periodontal bone resorption ↓; macrophage/neutrophil infiltration ↓; IL-1β/IL-6/IL-17/TNF-α ↓; IL-4/IL-10 ↑; IL-10/IL-17 ratio ↑.	EA can effectively alleviate inflammation and bone resorption in LIP mice, potentially via the regulation of myeloid cells, cytokines, and periodontal microbiome
Wang et al. [45]	2023	China	mechanistic animal study	Animal	Sham electroacupuncture	Persistent inflammatory pain (CFA-induced)	Electroacupuncture (Zusanli-specific)	Once daily for 21 consecutive days	Dense wave 2/100 Hz, 1 mA	15 minutes	ST36	ST36; SP6	Not reported (study focused on sympathetic sprouting rather than HRV metrics).	Not reported	CFA pain model: EA did not change TNF or IL-6 overall; CFA increased TNF/IL-6 and decreased IL-10; EA increased IL-10 in paw skin; antagonist blocked IL-10 increase.	DRG-focused mechanism: EA inhibited sympathetic–sensory coupling in ipsilateral L6 DRG; pathways implicated include TRPV1/CGRP, ERK, and TLR4 signaling (MEK/p-MEK, CREB/p-CREB; TLR4/IRF3/p-IRF3; P-65/p-P65).	EA inhibited mechanical and thermal pain hypersensitivities. EA inhibited neurogenic inflammation. Neurogenic inflammation was characterized by decreased expression of substance P, hyaluronic acid, bradykinin, and prostacyclin in colitis rat skin tissues corresponding to the L6 DRG. EA treatment reduced the activation of the TRPV1/CGRP signaling pathway in L6 DRG. CGRP was labeled to mark sensory nerve fibers. The sprouting of tyrosine hydroxylase-positive sympathetic fibers into sensory neurons was abrogated by EA.	Gut–brain axis findings	Electroacupuncture improves colitis-associated inflammation and referred somatic pain via sympathetic–sensory coupling at dorsal root ganglia.	Colitis-pain model: DAI decreased on days 11 and 14; TWL and MWT improved on days 11 and 14 vs sham-EA (figure-annotated significance).	Colitis referred pain: lesion reduction; reduced sympathetic-sensory coupling; reduced SP/HA/BK/PGI2; inhibited TRPV1/CGRP, ERK, TLR4 signaling.	DAI ↓; TWL ↑; MWT ↑; sympathetic–sensory coupling ↓; IL-1β/IL-6/TNF-α ↓; IL-10 ↑; SP/HA/BK/PGI2 ↓.	Electroacupuncture at ST36 relieved colitis-associated hyperalgesia by inhibiting neurogenic inflammation and sympathetic sprouting.
Tanaka et al. [82]	2023	Brazil, USA, Italy, and Canada	mechanistic animal study	Animal	Sham electroacupuncture and pharmacological blockade (FPR2/ALX antagonist)	Sepsis-induced cardiac dysfunction	Electroacupuncture	Once daily for 4 consecutive days	2/10 Hz; dense–disperse asymmetric balanced wave; intensity 0.6 mA	20 minutes	ST36; SP6	PC6	Not reported	Not reported	Sepsis cardiac dysfunction: EA decreased plasma TNF-α and IL-1β and reduced myocardial TNF-α/IL-1β; IL-6 and CRP not reported.	Analgesia discussed as primarily via central opioid peptides, but pain proximity suggests local/segmental contributions; EA inhibits pain through peripheral, spinal, and supraspinal pathways.	The effect depends on the activation of peripheral FPR2/ALX. EA-induced analgesia involves the release of endogenous opioids into the inflamed tissue, which activate receptors on peripheral nerve terminals. Involvement of other mediators, such as adenosine and endocannabinoids, and non-opioid receptors, such as receptors for endothelin and formyl peptide receptor 2. EA treatment increased CAT activity in the paw muscle, p = 0.0381. Pretreatment with FPR2/ALX antagonist prevented this effect induced by EA. ROS and products from oxidation of membrane lipids and phospholipids have pro-nociceptive actions via TRP channels, mainly TRPA1 and TRPV1.	HPA/stress axis effects	Hypothalamic–pituitary–adrenal activation contributes to electroacupuncture effects, with cortisol and annexin-A1 mediating negative feedback on CRH release.	Not reported (mechanistic study; primary behavioral signal was reduced paw withdrawal response frequency on day 1 (p = 0.0008); antagonist blocked effect (p < 0.0001)).	Cytokines (TNF, IL-6, IL-4, IL-10); antioxidants (catalase, SOD); oxidative stress markers; MPO activity.	Mechanical hyperalgesia ↓; IL-10 in paw skin ↑; catalase (CAT) activity ↑.	Electroacupuncture-mediated antihyperalgesia was partly dependent on IL-10 upregulation and antioxidant activity via FPR2/ALX signaling.
Wu et al. [83]	2023	China	mechanistic animal study	Animal	Sham operation control, vagotomy control, and sham acupuncture	Traumatic brain injury (controlled cortical impact model)	Electroacupuncture	Single session	2/15 Hz, 2 mA	20 minutes	PC6	PC6; GV20	EA increased vagus activity and reduced LF/HF in sepsis-induced cardiac dysfunction (LF/HF in EA-treated septic rats approximately 0.55).	Cardioprotection mediated via vagus-dependent cholinergic pathway; efferent vagus acetylcholine activates macrophages via α7nAChR, inhibiting pro-inflammatory cytokines; α7nAChR expression on cardiac macrophages increased with EA.	TBI model: IL-6, IL-1β, and TNF-α increased in model and decreased with acupuncture; IL-10 higher with treatment; CRP not reported.	Not reported	Not Reported	Not Reported	Not Reported	Sepsis cardiac dysfunction: EA at PC6 improved survival (median survival 5.5 vs 1.5 days, p < 0.05) and improved lactate (1.67 ± 0.20 vs 2.82 ± 0.26 mmol/L, p < 0.005).	Sepsis cardiac model: LF/HF decreased with EA; MAP maintained; EF reduced in sepsis vs control and improved with EA; BNP/cTnI increases attenuated by EA.	LVEF ↑; LF/HF ↓; lactate ↓; MAP ↑ (maintained); TNF-α and IL-1β ↓; survival rate ↑.	Electroacupuncture at PC6 protected against sepsis-induced cardiac dysfunction through vagal cholinergic mechanisms.
Zhang et al. [25]	2023	China	mechanistic animal study	Animal	Sham operation control and NF-κB inhibitor comparator	Atrial fibrillation	Electroacupuncture	Sparse (2 Hz), dense (100 Hz), sparse–dense (2/15 Hz or 2/100 Hz); intensity not reported	15 minutes	GV26; GB20; PC6	PC6	Not reported	Not reported	Not reported	TBI: EA promoted microglial M2 polarization via inhibition of NF-κB/COX2 pathway in peri-injury cortex, supporting neuroprotection.	EA promotes microglial polarization towards the M2 phenotype through the suppression of NF-κB/COX2 pathway, thus exerting neuroprotective effects after TBI. The characteristic of the M1 phenotype was the secretion of pro-inflammatory cytokines, such as IL-6, IL-1β, and TNF-α. The M2 phenotype releases anti-inflammatory cytokines, such as IL-10 and transforming growth factor β, to keep the immune system in balance. Both EA with 2/100 Hz and PDTC reduced the levels of p-NF-κB, COX2 and M1 markers and increased the levels of M2 markers. TBI significantly reduced Arg-1 levels, whereas EA increased Arg-1 levels. TUNEL was used to examine neuronal apoptosis.	Not Reported	Not Reported	TBI: 2/100 Hz EA improved neurological/cognitive outcomes; mNSS lower vs TBI at 3 and 7 days (p < 0.01) and MWM performance improved (p < 0.01).	TBI model: MDA/IL-6/IL-1β/TNF-α down; SOD/GSH-Px up; apoptosis reduced; p-NF-κB and COX2 reduced; microglia phenotype shifted (iNOS↓/Arg1↑) (p < 0.01 or p < 0.05).	mNSS ↓; escape latency ↓; platform crosses ↑; MDA ↓; SOD and GSH-Px ↑; IL-6/IL-1β/TNF-α ↓; IL-10 and Arg-1 ↑; TUNEL+ apoptosis ↓; p-NF-κB and COX2 ↓.	Electroacupuncture promoted neurological recovery after traumatic brain injury by suppressing neuroinflammation and promoting microglial M2 polarization.	
Su et al. [84]	2024	China	mechanistic animal study	Animal	Sham electroacupuncture and alternative acupoint comparator	Functional dyspepsia (disorder of gut–brain interaction)	Electroacupuncture via chronically implanted electrodes	Ten consecutive days	2 Hz, 2 mA; pulse duration 100 ms, interval 400 ms	20 minutes	PC6; LU9	ST36	AF model: SDNN differed (F(3,39) = 13.55; p < 0.0001). Sympathetic nerve frequency differed (F(2,16) = 8.190; p = 0.0036). Vagus nerve frequency differed (F(2,16) = 24.26; p < 0.0001). LF/HF differed (F(2,30) = 37.86; p < 0.0001).	Not reported	Functional dyspepsia model: TNF-α, IL-1β, and IL-6 elevated and significantly reduced with EA; IL-10 unchanged; CRP not reported (values provided in entry).	AF model: EA downregulated c-Fos in PVN, RVLM, and DMV and upregulated c-Fos in nucleus ambiguus; EA reversed AF-associated c-Fos increases in PVN/RVLM; IML contains preganglionic sympathetic neurons.	Not Reported	HPA/stress axis effects	The paraventricular nucleus integrates neuroendocrine, homeostatic, and stress responses relevant to acupuncture effects.	AF model: AF duration differed across groups (F(3,39) = 6.887, p = 0.0008); EA reduced AF incidence (40% vs 77%).	AF model: inducibility; HRV; cervical sympathetic/vagal nerve activity; c-Fos; P-wave duration; SDNN.	AF inducibility ↓; AF duration ↓; sympathetic nerve activity ↓; vagal nerve activity ↑; LF/HF ↓.	Electroacupuncture reduced atrial fibrillation susceptibility by restoring sympathetic–vagal balance.
Zhang et al. [85]	2024	China and USA	mechanistic animal study	Animal	Sham electroacupuncture without current	Myocardial infarction	Electroacupuncture	Acute, single session (1 hour in overnight-fasted rats)	25 Hz, 4 mA; train on-time 2 s, off-time 3 s	60 minutes	ST36	PC6	EA reduced plasma norepinephrine under stress versus sham: 1.18 ± 0.21 vs 2.54 ± 0.51 (p < 0.05).	CAP described (efferent vagus → splenic nerve → NE → ChAT+ T cells → ACh → α7nAChR). Proposed TEA pathway: peripheral nerve activation → NTS signaling → enhanced vagal efferents → acetylcholine release → α7nAChR-mediated suppression of TNF-α/IL-1β/IL-6 and improved gastric motility.	MI model: EA reduced TNF-α, IL-1β, and IL-6 and promoted macrophage M2 polarization; CRP not reported.	EA at ST36: peripheral nerve activation engages NTS neurons and CNS processing produces enhanced vagal efferent signal.	Not reported	Gut–brain axis findings	Functional gastrointestinal disorders represent disorders of gut–brain interaction; electroacupuncture enhances gastric motility.	Burn/GE correlation study: EA accelerated gastric emptying vs sham (70.6% vs 56.8%, p = 0.01) and related outcomes.	Sepsis/burn correlations: cytokines (TNF-α, IL-1β, IL-6, IL-10), NE, and correlations with GE.	Plasma TNF-α ↓; IL-1β ↓; IL-6 ↓; plasma NE ↓; GE ↑.	Electroacupuncture inhibited pro-inflammatory cytokine release in functional dyspepsia through cholinergic anti-inflammatory pathways.
Peng et al. [86]	2024	China	mechanistic animal study	Animal	Sham operation control and myocardial infarction model	CFA-induced inflammatory arthritis	Manual acupuncture	EA course with outcomes assessed at 7 and 28 days (session schedule variably described)	2/15 Hz, 1 mA	20 minutes	PC6	ST36	MI increased LF and LF/HF and decreased HF; EA decreased LF and LF/HF and increased HF (significant vs MI).	Not reported	CFA arthritis model: Rap1, IL-1β, TNF-α, and p-p65 reduced with MA; CRP and IL-6 not reported.	Central sympathetic involvement flagged as an area for further investigation.	Not Reported	Not Reported	Not Reported	MI model: EA improved systolic function (EF and FS, both p < 0.0001 vs MI).	MI remodeling: fibrosis reduced; TH and GAP43 reduced; M2 polarization promoted; NE increase slowed; LF and LF/HF reduced and HF increased.	FS ↑; EF ↑; myocardial fibrosis ↓; TH and GAP43 ↓; NE ↓; macrophage M2 polarization ↑; LF/HF ↓.	Electroacupuncture attenuated post-myocardial infarction sympathetic remodeling and improved cardiac function via macrophage polarization.
Xu et al. [33]	2024	China	mechanistic animal study	Animal	Sham manual acupuncture at non-acupoint	Ischemic stroke (middle cerebral artery occlusion)	Electroacupuncture	3 consecutive days	Not reported	30 minutes	ST36	GV20	Not reported	Not reported	Ischemic stroke (MCAO): EA down-regulated Il-6, Tnf-α, Il-1β, and Ccl-2 mRNA in cortex; CRP not reported (approximate fold-changes noted).	Not reported	MA can alleviate inflammatory pain by inhibiting the NF-κB signaling pathway via upregulating A3R expression of the superficial fascia of the ST36 acupoint site in CFA-induced arthritis rats. Furthermore, after MA, the mRNA and protein expression of A3R was upregulated in CFA-induced arthritis rats. In contrast, the protein levels of Rap1, and p-p65 were downregulated after MA. The protein levels of Rap1, IL-1β, TNF-α, and p-p65 were significantly decreased in the CFA + MA group compared with the CFA group, p < 0.01. Compared with the CFA + MA group, the protein levels of IL-1β and TNF-α were significantly increased in the CFA + MA + Reversine group, p < 0.05.	Not Reported	Not Reported	MA analgesia: MA reduced allodynia and increased PWL/PWT vs CFA (p < 0.01 across days; day-specific p-values as reported).	CFA+MA: ankle swelling reduced (p < 0.01); rearing and ambulatory distance improved (p < 0.01); A3R antagonist worsened swelling post-MA.	PWL ↑; PWT ↑; ankle swelling ↓; A3R expression ↑; TNF-α/IL-1β/Rap1/p-p65 ↓.	Manual acupuncture reduced inflammatory pain through NF-κB inhibition mediated by A3 adenosine receptor activation.
Ren et al. [24]	2024	China	mechanistic animal study	Animal	Sham operation control and pharmacological antagonist comparator	Postoperative pain following thoracoscopic surgery	Transcutaneous electrical acupoint stimulation (TEAS)	Up to 3 days (immediate post-reperfusion, then at 24-hour intervals)	Dense–disperse wave 4/20 Hz; voltage 1–3 V; current 1–3 mA	20 minutes	GV26; GV20	LI4; PC6	Not reported	Not reported	Thoracoscopic surgery: IL-6 measured and lower in treatment group at early timepoints, returning to baseline by 24 h; other markers not reported.	Ischemic stroke: EA inhibited neuroinflammation by suppressing TRPV4 and reducing M1 polarization of microglia/macrophages, improving neurologic deficit scores.	Electroacupuncture ameliorates neuroinflammation by inhibiting TRPV4 channel in ischemic stroke. EA may inhibit neuroinflammation and exhibit a protective effect against ischemic brain injury by suppressing TRPV4 and the subsequent M1 polarization of microglia/macrophages. EA reduced the elevated expression of TRPV4 following MCAO. EA significantly inhibited the activated microglia/macrophages and the polarization to the pro-inflammatory phenotype M1 in the ischemic cerebral cortex. The mechanism involves inhibition of the TRPV4 channel to suppress M1 polarization in microglia/macrophages following ischemic stroke in mice.	Not Reported	Not Reported	MCAO mice: EA reduced infarct volume vs MCAO (approx. 58 mm³ vs 26 mm³; p < 0.01).	Stroke/TRPV4: retention time increased; neurodeficit reduced (e.g., day 1 score 4.7 vs 2.3, p < 0.001); microglia/macrophage M1 polarization inhibited; Tnf-α/Il-6/Ccl-2 mRNA suppressed; EA and comparator inhibitor arms similar.	Neurological deficit score ↓; cerebral infarction volume ↓; rotarod retention time ↑; microglia/macrophage activation ↓; TRPV4 expression ↓; M2 markers: no statistically significant difference.	Electroacupuncture protected against ischemic brain injury by suppressing TRPV4-mediated microglial activation.
Liu et al. [35]	2024	China	RCT	Human	Inactive electrical stimulation control	Chronic fatigue syndrome	Traditional needle acupuncture	Two perioperative sessions (30 minutes pre-induction; 30 minutes post-surgery)	Dense–disperse wave 2/200 Hz; intensity adjusted to De Qi tolerance	30 minutes	PC6; LI4	ST36	Not reported	Not reported	Not reported	PC6 and LI4 may influence frontal cortex to participate in ventrolateral thalamic nucleus pain modulation.	Patients in Group T also had higher β-EP content. β-EP is an endogenous analgesic substance released by the pituitary chiefly, which can inhibit pain and reduce stress level. Endogenous opioids evoked by electroacupuncture are frequency dependent and have been proven to play a central role in the pain inhibition.	HPA/stress axis effects	β-endorphin release contributes to pain inhibition and stress reduction; transcutaneous electroacupuncture also modulates stress responses.	Primary outcome postoperative pain intensity (NRS): AUC pain 6–72 h lower in TEA group vs control (268.7 ± 56.1 vs 324.8 ± 66.7, p < 0.01).	Perioperative analgesia: remifentanil lower in TEA group (p < 0.01); rescue analgesia lower (85.7% vs 59.5%, p < 0.01); PONV lower (35.7% vs 14.3%, p = 0.023); satisfaction higher (p < 0.01).	Postoperative pain (NRS/AUC) ↓; remifentanil ↓; rescue analgesia demand ↓; PONV ↓; satisfaction ↑; β-EP ↑; IL-6 ↓.	Perioperative transcutaneous electroacupoint stimulation reduced postoperative pain, analgesic use, and nausea while improving patient satisfaction.
Li et al. [87]	2025	China	RCT	Human	Sham acupuncture control	Major depressive disorder	Intradermal acupuncture	Ten treatments, every other day	Not reported	15 minutes	ST36; CV4	PC6; SP6; LR3	CFS/qi deficiency: HRV total negatively correlated with QSS (p < 0.01); HF positively correlated with QSS (p < 0.01). LF/HF defined as log(LF/HF).	Immediate effects suggest acupuncture can stimulate vagal (parasympathetic) fibers and reduce heart rate.	Not reported	Central control: heart rate regulation influenced by higher centers including DMV and nucleus ambiguus in medulla oblongata; acupuncture can influence ANS function at CNS level.	Not Reported	Not Reported	Not Reported	HRV total increased significantly after first treatment in groups C–F (all p < 0.001 vs baseline).	Fatigue/Qi outcomes: QSS, CFS score, SF-36; Guanyuan vs Zusanli effects on LF/HF and HF (p < 0.05).	Mean HR ↓; HRV total ↑; LF ↑; HF ↑.	Dual-point acupuncture targeting Zusanli and Guanyuan produced superior autonomic regulation compared with single-point stimulation.
Wu et al. [88]	2025	China	RCT	Human	Pharmacological comparator (SSRIs) and sham acupuncture	Chronic hypoxia–induced cognitive impairment	Electroacupuncture	Ten sessions over 6 weeks	Not reported	Not reported	HT7; PC6; SP6; LR3	ST36; GV20	Not reported (autonomic modulation proposed; no HRV metrics).	Intradermal acupuncture at PC6 may modulate sympathetic/vagal balance via median nerve stimulation; reduced palpitations/fatigue/nausea attributed to autonomic modulation (mechanistic inference).	Chronic hypoxia: EA reduced brain TNF-α mRNA; contextual discussion includes TNF-α/IL-1β/IL-6/CCL2 as M1 microglia products.	Intradermal acupuncture MRI: altered functional connectivity between striatum and frontal/cerebellar regions; implicated dopamine reward and 5-HT systems; ROIs included striatum, VTA, DRN, and MRN; IA may exert antidepressant effects via striatal connectivity modulation.	Not reported.	Not Reported	Not Reported	Depression (HAMD-17): SSRIs+AIA reduced scores more than SSRIs+SIA (MD −4.9, p < 0.001) and SSRIs alone (MD −5.1, p < 0.001).	Depression trial: SDS and PSQI; HAMD-17 factor scores improved (anxiety/somatization, retardation); adverse events.	HAMD-17 reduction ↑ (greater reduction in AIA group); SDS reduction ↑; palpitations/somnolence/nausea ↓.	Combined acupuncture intervention with SSRIs enhanced antidepressant efficacy, reduced adverse effects, and modulated reward-related brain circuits.
Wan et al. [89]	2025	China	mechanistic animal study	Animal	Disease model control (chronic hypoxia; no sham control reported)	Sepsis	Manual acupuncture and electroacupuncture	Once daily with a break every 6 days for a total of 14 days	Not reported	30 minutes	GV20; GV24; ST36	ST36	Not reported	Not reported (focus on neuroinflammation/glial polarization).	Multiple compartments: TNF-α and IL-6 decreased in brain/serum/lung; IL-1β decreased; nitrite/HMGB1/NO/MPO/iNOS decreased; α7nAChR increased; CRP not reported.	Chronic hypoxia model: EA reduced neuronal damage/apoptosis/myelin loss in cortex and hippocampus and inhibited overactivation of microglia and astrocytes; A1 astrocytes release cytokines including TNF-α and IL-6, contributing to neuroinflammation; ST36 referenced as engaging limbic system/default mode network.	EA suppressed the expression of Iba1 and GFAP, markers of microglial and astrocytic activation, respectively. EA promoted the polarization of microglia towards the M2 anti-inflammatory phenotype by downregulating iNOS and upregulating Arg1. EA reduced the expression of C3, a marker of A1 astrocytes, thereby preventing astrocytic polarization towards the pro-inflammatory state. EA reduced neuronal damage and apoptosis.	Not Reported	Not Reported	Not reported (mechanistic study; behavioral outcomes improved in anxiety-like behavior and memory tests with EA vs model as reported).	Neuroprotection: reduced neuronal damage/apoptosis; reduced glial overactivation; M2 polarization (iNOS↓, Arg1↑); TNF-α reduced.	OFT distance ↑; center-zone time ↑; Y-maze alternation ↑; MWM platform crosses ↑; TNF-α mRNA ↓; iNOS mRNA ↓; Arg1 mRNA ↑; C3 mRNA ↓.	Electroacupuncture improved cognitive function under chronic hypoxia by reducing neuroinflammation and glial overactivation.
Zeng et al. [22]	2025	China	literature review of rodent studie	Animal	Multiple mechanistic controls (vagotomy, genetic ablation, pharmacological blockade)	Alzheimer’s disease	Electroacupuncture	Varied by study design: single pretreatment vs repeated sessions	Varies among reviewed studies	Varied across reviewed studies	ST36	ST36	Catecholamines increased (serum NA, A, DA); c-Fos increased, including in DMV (no HRV metrics).	Acupuncture mechanisms include vagus-linked pathways (CAP; vagal–splanchnic axis) and sympathetic pathways; acetylcholine–α7nAChR signaling implicated; EA alleviated LPS-induced ARDS via α7nAChR-mediated inhibition of ferroptosis.	Neuroinflammation model: EA reduced hippocampal IL-1β and TNF-α; IL-6 not altered in one analysis (other section noted IL-6 suppression in EA/hM3D groups); CRP not reported.	Brain: NSE decreased; ST36-to-NTS signaling described with downstream activation of DMV; EA may also activate spinal sympathetic axis and produce vagal output.	Not reported. General pathways affected include: modulating the MAPK and NF-κB signaling pathways; regulating the NLRP3 inflammasome. Spleen: NF-κB p65 ↓. Colon: TLR4 ↓, MyD88 ↓, p–NF–κB/NF–κB ↓. Lung: caspase 1 ↓, NLRP3 ↓.	Gut–brain axis findings; HPA/stress axis effects	Sepsis disrupts intestinal barrier integrity; electroacupuncture improves gut permeability markers and modulates hypothalamic–pituitary–adrenal–mediated immune regulation.	Not Reported	Not reported (general sepsis-lung/kidney/cardiac biomarker claims without study-specific quantitative secondary outcome reporting).	Injury/pathology scores ↓; TNF-α/IL-6/IL-1β/HMGB1 ↓; BUN/Cr/NSE ↓; catecholamines (NA, A, DA) ↑.	Acupuncture at ST36 alleviated multi-organ dysfunction in sepsis by reducing tissue injury, edema, and inflammatory burden.
Yu et al. [27]	2025	China	mechanistic animal study	Animal	Sham electroacupuncture at non-acupoint and no-treatment control	Neurological motor and excitability disorders	Electroacupuncture and manual acupuncture	Every other day for 1 month	2 Hz, 2 mA; pulse width 300 μs	30 minutes	ST36; ST37	LI4; PC6; ST36; LR3; GV20; GB34; CV6	Not reported	Neural circuitry remains incompletely defined; vagal afferent dependence supported by ablation reversing EA effects; EA at ST36/ST37 activates NTS–LC noradrenergic circuitry via vagal afferents.	Not reported	AD model: EA at ST36/ST37 improved cognition via gastric vagal afferent-mediated activation of NTS–LC noradrenergic circuit; increased c-Fos in NTS and increased TH+/c-Fos+ neurons in LC; LC-NE system implicated in neuroinflammation and synaptic ultrastructure preservation.	This intervention suppressed proinflammatory cytokines expression and microglial hyperactivation. EA markedly decreased the number of activated microglia in the hippocampal CA1 region compared with the model and sham EA groups, p < 0.01. The mechanism involves ADRB2/PKA/CREB pathway modulation. β2-AR activation by NA or β2-AR agonists can trigger the cAMP/PKA/CREB pathway in microglia, thereby suppressing microglial activation and alleviating neuroinflammation.	Gut–brain axis findings	Electroacupuncture at ST36 and ST37 improves cognition via gastric vagal afferent activation of the NTS–LC noradrenergic circuit, supporting a gut–brain vagal axis mechanism.	Cognition (AD model): EA improved acquisition latencies from day 3 onward and increased target-quadrant dwell time vs model/sham (p < 0.01).	AD model: discrimination index improved (p < 0.01); spine density increased (p < 0.05); mushroom spines increased (p < 0.05); TH⁺/c-Fos⁺ in LC increased (p < 0.01); ADRB2/PKA/CREB signaling upregulated (p < 0.01).	Escape latency ↓; target quadrant dwell time ↑; recognition index ↑; dendritic spine density ↑; CA1 microglial activation ↓; IL-1β/TNF-α/IL-6 ↓; ADRB2/p-PKA/p-CREB ↑.	Electroacupuncture improved learning, memory, synaptic plasticity, and reduced neuroinflammation in experimental cognitive impairment.
Liu et al. [28]	2025	Australia	SLR and MA	Human	sham acupuncture, no treatment or usual training.	Focus on modulating brain excitability and motor function. Patients with neurological disorders, including stroke, spinal cord injuries and Parkinson’s disease.	Electroacupuncture (EA) and manual acupuncture (MA).	Not reported (highly variable across 34 included studies)	Not reported	Not reported	LI4; ST36; LI11; TE5; GB34	LI4,PC6,ST36, LR3, GV20, GB34, CV6	Not reported	Not reported	Not reported	CSE/brain excitability: EA proposed to enhance corticospinal excitability via sensory nerve activation transmitting to CNS and promoting neural plasticity; spinal reflex mechanisms may indirectly modulate motor neuron activity.	Acupuncture may promote or inhibit the release of neurotransmitters such as serotonin and endorphins, which can modulate neuronal excitability. Endogenous opioids evoked by electroacupuncture are frequency dependent.	Not Reported	Not Reported	Primary outcome corticospinal excitability (MEPs): acupuncture increased CSE; EA showed larger effect than MA (effect sizes 0.53 mV vs 0.43 mV; both p < 0.00001).	Meta-analysis: safety/adverse events; heterogeneity metrics; mean MEPs in pathological and healthy cohorts; 47% of studies reported adverse events (16/34); one study reported mild bleeding/hematoma.	CSE/MEP amplitude ↑.	Systematic evidence indicates both electroacupuncture and manual acupuncture safely enhance corticospinal excitability and motor function.
